# Parathyroid Hormone Promotes Human Umbilical Vein Endothelial Cell Migration and Proliferation Through Orai1-Mediated Calcium Signaling

**DOI:** 10.3389/fcvm.2022.844671

**Published:** 2022-03-16

**Authors:** Shuhao Wang, Lijie Xu, Yv Wu, Hailong Shen, Zhangying Lin, Yang Fang, Lesha Zhang, Bing Shen, Yehai Liu, Kaile Wu

**Affiliations:** ^1^Department of Otorhinolaryngology, Head and Neck Surgery, The First Affiliated Hospital of Anhui Medical University, Hefei, China; ^2^Department of Otorhinolaryngology, General Hospital of Anhui Wanbei Coal Power Group, Suzhou, China; ^3^Department of Physiology, School of Basic Medical Sciences, Anhui Medical University, Hefei, China

**Keywords:** secondary hyperparathyroidism, parathyroid hormone, store-operated Ca^2+^ entry, human umbilical vein endothelial cells, COL1A1, NFAT

## Abstract

Parathyroid hormone is the main endocrine regulator of extracellular calcium and phosphorus levels. Secondary hyperparathyroidism–induced endothelial dysfunction may be related to calcium homeostasis disorders. Here, we investigated the effects of parathyroid hormone on human umbilical vein endothelial cells (HUVECs) and characterized the involvement of store-operated Ca^2+^ entry (SOCE) and the nuclear factor of activated T cells (NFAT) signaling pathway. We used immunoblot experiments to find that parathyroid hormone significantly enhanced the expression of the Orai1 channel, a type of channel mediating SOCE, SOCE activity, and Orai1-mediated proliferation of HUVECs but did not increase Orai2 and Orai3. RNA-seq was utilized to identify 1,655 differentially expressed genes (823 upregulated and 832 downregulated) in parathyroid hormone–treated HUVECs as well as enhanced focal adhesion signaling and expression levels of two key genes, namely, COL1A1 and NFATC1. Increased protein and mRNA expression levels of COL1A1 and NFATC1 were confirmed by immunoblotting and quantitative RT-PCR, respectively. Cytosol and nuclei fractionation experiments and immunofluorescence methods were used to show that parathyroid hormone treatment increased NFATC1 nuclear translocation, which was inhibited by a calcineurin inhibitor (CsA), a selective calmodulin antagonist (W7), an Orai channel inhibitor (BTP2), or Orai1 small interfering RNA (siRNA) transfection. Parathyroid hormone also increased COL1A1 expression, cell migration, and proliferation of HUVECs. The PTH-induced increase in HUVEC migration and proliferation were inhibited by CsA, W7, BTP2, or COL1A1 siRNA transfection. These findings indicated that PTH increased Orai1 expression and Orai1-mediated SOCE, causing the nuclear translocation of NFATC1 to increase COL1A1 expression and COL1A1-mediated HUVEC migration and proliferation. These results suggest potential key therapeutic targets of Orai1 and the downstream calmodulin/calcineurin/NFATC1/COL1A1 signaling pathway in parathyroid hormone–induced endothelial dysfunction and shed light on underlying mechanisms that may be altered to prevent or treat secondary hyperparathyroidism–associated cardiovascular disease.

## Introduction

Cardiovascular disease (CVD) is the leading cause of death among patients with chronic kidney disease (1). Owing to kidney disease, immune functions decline and various bodily functions are impaired, often accompanied by a variety of complications. A common complication of chronic kidney failure is secondary hyperparathyroidism (SHPT). SHPT is an independent risk factor for cardiovascular events and death in patients, seriously affecting survival rates and patient quality of life. SHPT is clinically characterized by parathyroid hyperplasia and excessive secretion of parathyroid hormone (PTH) (2–4). Elevated PTH may cause severe bone damage and the occurrence of CVD. Vascular dysfunction is a key link in the occurrence of CVD. Although the pathogenesis of this dysfunction is not fully understood, endothelial dysfunction is considered to be a crucial factor in this process (5, 6).

PTH is an 84 amino acid polypeptide hormone that mainly functions as a mediator of bone remodeling and is an important regulator of calcium (Ca^2+^) homeostasis (5). Increased intracellular Ca^2+^ concentration ([Ca^2+^]_i_) regulates various functions of cells, including growth, proliferation, differentiation, migration, and death. PTH receptors are distributed throughout the cardiovascular system, including on smooth muscle cells, endothelial cells, and cardiomyocytes (7, 8). Several studies have reported that endothelial dysfunction associated with SHPT may be related to the dysfunction of Ca^2+^ homeostasis in endothelial cells (9, 10). Store-operated Ca^2+^ entry (SOCE) activity plays an important role in the occurrence and development of CVD. In SOCE, G-protein coupled receptor activation generates phospholipase C and subsequent inositol triphosphate (IP_3_) production. IP_3_ acts on IP_3_ receptors located in the endoplasmic reticulum membrane to induce Ca^2+^ release from Ca^2+^ stores. Eventually, Ca^2+^ stores are depleted and this depletion activates stromal interaction molecule 1 (STIM1) to polymerize and move close to the cell plasma membrane to activate Orai channels, which mediate Ca^2+^ influx to replenish the exhausted Ca^2+^ stores. There are three types of Orai channels, Orai1, Orai2, Orai3, and two types of STIMs, STIM1 and STIM2. The Orai1 channel is activated by STIM1 (11, 12). Sustained SOCE activity is critical for activating nuclear factor of activated T cells (NFAT) in T lymphocytes, a family of transcription factors that was initially thought to regulate the expression of early immune response genes. NFAT is composed of five family members: NFATC5 is regulated by osmotic pressure, and NFATC1-4 is mainly regulated by the calcium-calcineurin signaling pathway. At rest, NFAT exists in the cytoplasm in a phosphorylated state that can be translocated to the nucleus for transcriptional regulation after activation and dephosphorylation. Although it has been reported that SOCE activity is critical for the activation of the NFAT signaling pathway in endothelial cells, the role this pathway plays in the effects of PTH on endothelial cells is unclear (13–15).

Collagen type I α 1 (COL1A1) is a member of collagen family and collagen is the most abundant protein of extracellular matrix (ECM) being naturally present in human tissues such as skin, bones, cartilage, tendon and ligaments. This biopolymer interacts with cells and regulates cell anchorage, migration, proliferation and survival (16, 17). In the cardiovascular system, COL1A1 and its related signaling pathways are primarily associated with cardiac development and a variety of diseases processes. Furthermore, previous studies have shown that Ca^2+^ can regulate the expression of COL1A1 through the calcineurin/NFAT pathway in chondrocytes and cardiovascular system, involving in related diseases development and progression (18, 19). Therefore, endothelial damage may be associated with COL1A1 and the calcineurin/NFAT pathway signaling pathway.

In the present study, using pharmacological and molecular tools as well as functional assays, we elucidated the contribution of SOCE activity to PTH-induced intracellular Ca^2+^ mobilization and downstream cytokine production, providing a new potential target and theoretical basis for the treatment and prevention of CVD caused by SHPT.

## Materials and Methods

### Cell Culture and SiRNA Transfection

HUVECs were purchased from Guandao Biotechnology Co., LTD (Shanghai, China) and cultured in RPMI-1640 medium (Biological Industries, Israel) supplemented with 10% fetal bovine serum, 100 U/mL penicillin G, and 100 U/mL streptomycin sulfate at 37°C in a humidified atmosphere containing 5% CO_2_. For experiments, HUVECs were cultured in media containing different concentrations of PTH (C600082, Sangon Biotech, Shanghai, China) for 24 h.

HUVECs were seeded in 12-well plates and grown to 60% confluence for transfection. The cells were then transfected with Orai1 siRNA (200 nM) using lipofectamine 3000 and Opti-MEM (31985088, Thermo Fisher Scientific, USA) according to the manufacturer's instructions. Scrambled siRNA was used as a negative control. The siRNA sequence against human Orai1 (5′-GCACAGAUACCCAGAACUUUU-3′) was chemically synthesized by Biomics Biotechnology Co., LTD (Shanghai, China).

### Western Blotting

For fractionation experiments, the separation and preparation of cytoplasmic and nuclear extracts from HUVECs were conducted according to the manufacturer's protocol (P0028, Beyotime, Shanghai, China). The extracted proteins were separated by sodium dodecyl–sulfate polyacrylamide gel electrophoresis using 10% gels and transferred to polyvinylidene fluoride membranes. After being blocked in 5% skim milk, the membranes were incubated with primary antibodies, including rabbit anti-pNFATC1 (1:1,000, Affinity Biosciences, China), rabbit anti-NFATC1 (1:500, Affinity Biosciences, China), mouse anti-Lamin B1 (1:5,000, Affinity Biosciences, China), and rabbit anti-tubulin (1:5,000, Affinity Biosciences, China).

For immunoblot experiments, total protein was extracted from HUVECs. Protein-transferred membranes were incubated with the primary antibodies rabbit anti-Orai1 antibody, rabbit anti-Orai2 antibody, rabbit anti-Orai3 antibody (1:1,000 dilution; Affinity Biosciences, China) and rabbit anti-STIM1 antibody (1:1,000 dilution; Santa Cruz Biotechnology, USA) at 4°C for 24 h. The proteins were then treated with goat anti-rabbit IgG horseradish peroxidase–conjugated secondary antibody (1:5,000 dilution, Elabscience Biotechnology, China) at room temperature for 1 h. The protein signal was detected using an ECL detection system (Peiqing Technology, China). The optical density of each blot was normalized to that of β-tubulin or GAPDH and expressed as relative optical density. The blot images were analyzed using ImageJ (National Institutes of Health, Bethesda, Maryland).

### Quantitative Real-Time PCR (qPCR)

Total RNA was isolated using Trizol reagent (AC0101-B, SparkJade, China) according to the protocol supplied by the manufacturer. The concentration and quality of samples were analyzed using a Nanodrop 1000 system. Afterwards, cDNA was synthetized using the appropriate RNA with the TaqMan® Reverse Transcription kit (AG0304-B, SparkJade, China). Quantitative real-time PCR was performed using Taqman Gene Expression PCR Master Mix (AH0104-B, SparkJade, China). Reactions were performed in triplicate, and relative changes in gene expression were normalized to GAPDH as an internal control.

### [Ca^2+^]_i_ Measurement

[Ca^2+^]_i_ measurements were performed as previously described (20). Briefly, HUVECs were seeded on round glass cover slips placed in 12-well plates and treated with PTH (100 pM) for 24 h before measurement. The cells were incubated for 20 min at 37°C with 2 μM Fluo-8/AM and 0.02% pluronic F-127 (Invitrogen) in the culture media. Intracellular Ca^2+^ stores were depleted using 2 μM TG in a Ca^2+^-free saline solution (OPSS, 140 mM NaCl, 5 mM KCl, 2 mM CaCl_2_, 1 mM MgCl_2_, 10 mM glucose, and 5 mM HEPES, pH 7.3 to 7.4 adjusted with NaOH). Application of 2 mM Ca^2+^ to the medium evoked Ca^2+^ influx. The [Ca^2+^]_i_ is shown as fluorescence signals and was measured using a fluorescence microscope (Nikon T200, Tokyo, Japan). The baseline before the application of extracellular Ca^2+^ was considered F0, and [Ca^2+^]_i_ changes are expressed as the ratio of fluorescence relative to the intensity at baseline (i.e., F1/F0).

### Immunofluorescence Assay

HUVECs were seeded on coverslips for 24 h, grown to 60% confluence, and fixed with 4% paraformaldehyde. After being washed with phosphate-buffered saline (PBS), the cells were permeabilized and blocked with 0.2% Triton X-100 and 3% bovine serum albumin for 1 h at room temperature. Then, cells were incubated with a mouse polyclonal anti-NFATC1 antibody (1:100 dilution, Santa Cruz, USA) at 4°C overnight. The next day, cells were washed with PBS and incubated with an Alexa Fluor 488 secondary antibody (1:250 dilution, Thermo Fisher Scientific, USA) for 2 h at room temperature. The nucleus was counterstained with DAPI (4′, 6-diamidino-2-phenylindole). The sections were analyzed using confocal microscopy (Zeiss, Germany).

### Cell Viability Assay

HUVECs were seeded in 96-well plates and incubated at 37°C for 24 h. Cell viability was measured using a cell counting assay kit (CCK-8, Dojindo Molecular Technologies, MD, Japan) according to the manufacturer's instructions. Cells were treated with 100 pM PTH or underwent Orai1 siRNA transfection for 24 h before 10 μL of CCK-8 solution was added to each well. After 2 h at 37°C, absorbance at a wavelength of 450 nm was measured with an automatic enzyme-labeling instrument (Rayto, Shandong, China).

### Migration Assays

HUVECs were seeded in 12-well plates at a density of 2.0 ×10^5^/cm^2^ and cultured to confluent monolayers for 24 h. For *COL1A1* siRNA experiments, the cells were pre-transfected with siRNA for 24 h. A 200-μL pipette tip was used to create scratch “wounds.” The cells were washed with PBS twice to remove cell debris and incubated in the control medium containing PTH (100 pM) for 24 h. Scratch-wound healing was then observed, and images of the same location along the scratched edges were captured at 0 and 24 h. ImageJ was used to measure the healed area of the scratch and to quantitatively assess the speed of cell migration.

### Transcriptome Sequencing and Data Analysis

HUVECs were treated with PTH (100 pM) for 24 h, and four biological repeats were used in the experiments. After RNA extraction, HUVEC transcripts were obtained by cDNA library construction and sequencing. Fast QC software (21) was used to evaluate sequence quality. The index was established using Bowtie software, and transcript expression levels of samples from each of the four groups were analyzed to compare differences between each of two groups by using the edgeR package (22). The Metascape database was used to analyze and annotate enrichment of the functional pathways for the proteins encoded by the DEGs (21). KEGG pathway analysis of the DEGs was carried out in the same database.

### Statistical Analysis

Data are expressed as means ± SEM, and all data were analyzed using GraphPad Prism, version 7 software (GraphPad Software, San Diego, California). Statistical evaluation of the data was performed by using the unpaired Student's *t*-test and one way analysis of variance followed by Dunnett's multiple comparisons test. A two-sided value of *P* < 0.05 was considered statistically significant.

## Results

### Effects of PTH on SOCE-Related Protein Expression and Proliferation of Human Umbilical Vein Endothelial Cells (HUVECs)

**Figure 1:**
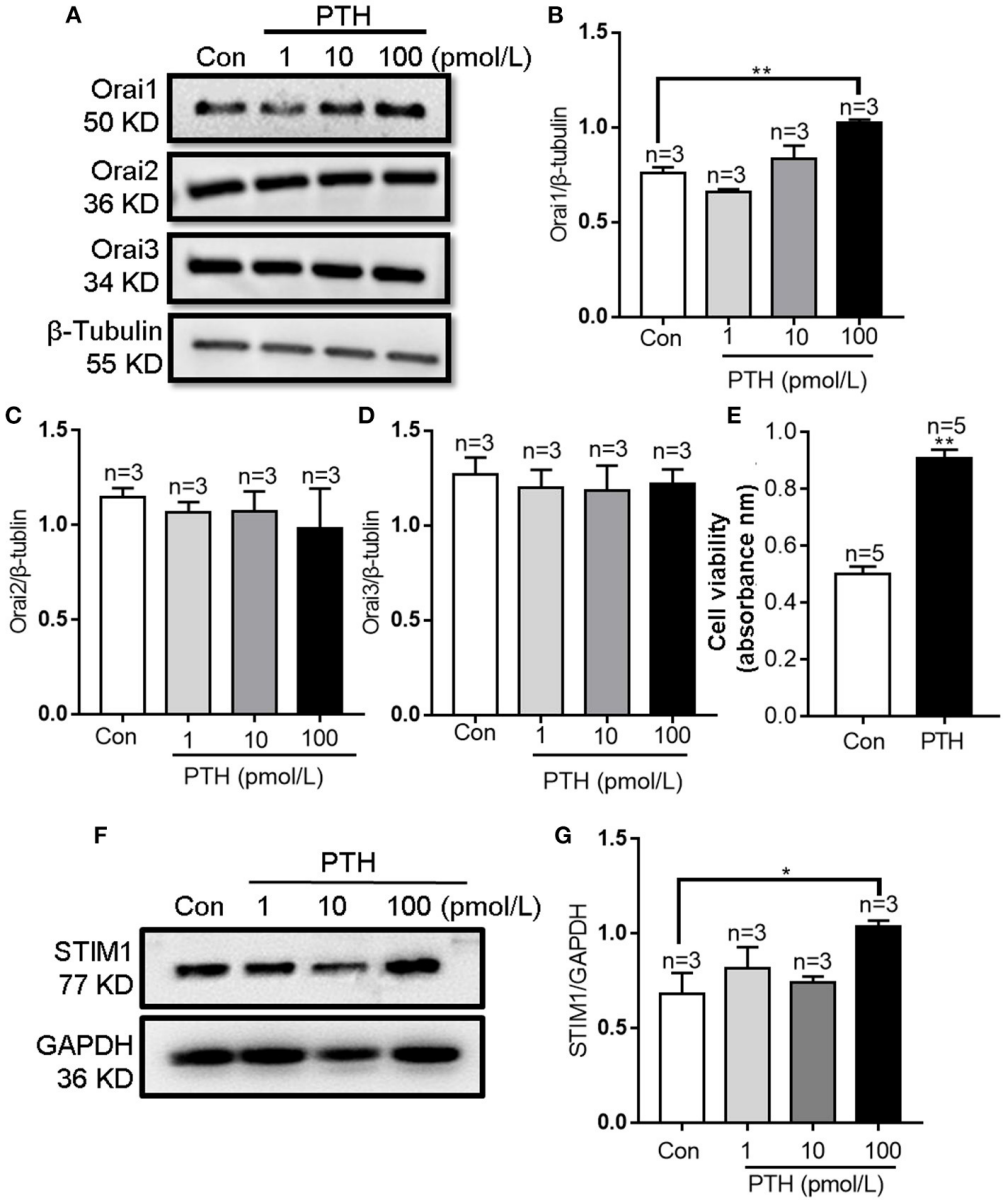
Effects of parathyroid hormone (PTH) on Orai expression and proliferation of human umbilical vein endothelial cells (HUVECs). **(A–D)** Representative Western blot images **(A)** and summary data **(B–D)** showing Orai1, Orai2, and Orai3 expression level changes in HUVECs after PTH treatment (1, 10, 100 pM) for 24 h compared with the vehicle control (Con). **(E)** Summary data showing viability of HUVECs treated with PTH (100 pM) for 24 h. **(F,G)** Representative Western blot images **(F)** and summary data **(G)** showing STIM1 expression level changes in HUVECs after PTH treatment (1, 10, 100 pM) for 24 h compared with the vehicle control (Con). Data are shown as the mean ± SEM; *n* = 3–5. *P < 0.05, **P < 0.01 vs. Con analyzed by one-way analysis of variance followed by Dunnett's multiple comparisons test.

After treating HUVECs with different PTH concentrations (1, 10, or 100 pM) for 24 h, we used Western blotting to investigate the change in the expression levels of Orais, which are major components of the channel in SOCE. Our results showed that treatment with PTH at a concentration of 100 pM, but not 1 or 10 pM, significantly increased the protein expression level of Orai1 (Figures 1A,B) and STIM1 (Figures 1F,G). By contrast, Orai2 and Orai3 protein expression levels were not affected by any tested concentration of PTH (Figures 1A,C,D). In addition, the expression of PTH receptor was not affected by PTH treatment in HUVECs (Supplementary Figure 1). The viability of HUVECs was determined using the CCK-8 assay. Compared with controls, treatment of PTH at 100 pM also enhanced the proliferation of HUVECs (Figure 1E).

### Role of Orai1-Mediated Ca^2+^ Influx in PTH-Induced HUVEC Proliferation

**Figure 2:**
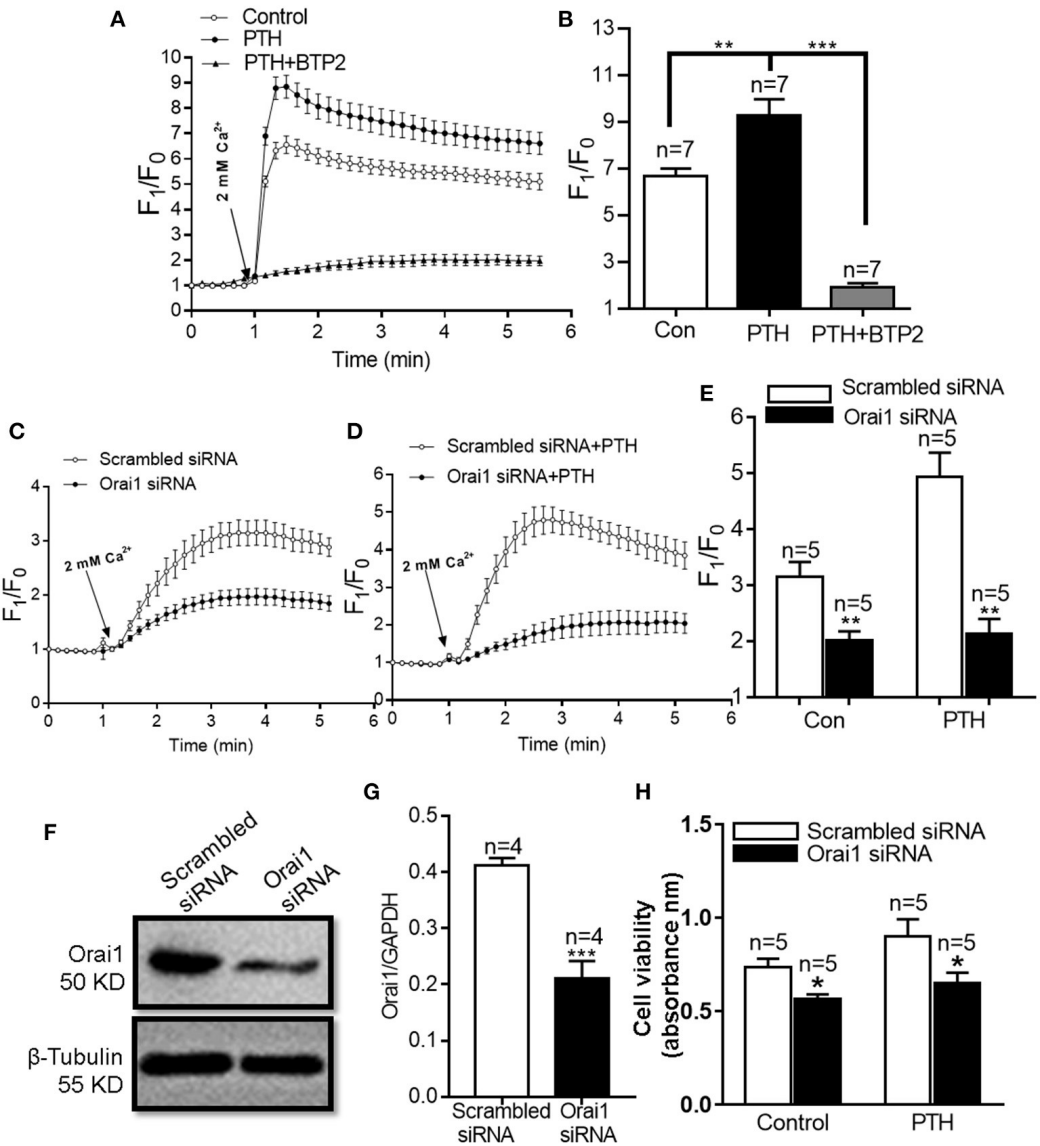
Effect of parathyroid hormone (PTH) on store-operated Ca^2+^ entry (SOCE) in human umbilical vein endothelial cells (HUVECs). **(A,B)** Representative traces **(A)** and summary data **(B)** showing thapsigargin (TG)-evoked SOCE in HUVECs after treatment with vehicle control, PTH (100 pM) or PTH plus BTP2 (an Orai nonspecific inhibitor) for 24 h. After depletion of the intracellular Ca^2+^ stores by treatment with 2 μM TG for 10 min in a Ca^2+^-free medium, SOCE was evoked by 2 mM Ca^2+^ application to the medium. Data are shown as the mean ± SEM; *n* = 7. **P < 0.01, ***P < 0.001 vs. Con or PTH. **(C–E)** Representative traces **(C,D)** and summary data **(E)** showing TG-evoked SOCE in HUVECs transfected with scrambled siRNA or Orai1 siRNA and treated with vehicle control or PTH (100 pM) for 24 h. **(F,G)** Representative images **(F)** and summary data **(G)** showing the effect of scrambled siRNA or Orai1 siRNA transfection on Orai1 expression in HUVECs. GAPDH was used as a loading control. **(H)** Summary data showing viability of HUVECs transfected with scrambled siRNA or Orai1 siRNA and treated with vehicle control or PTH (100 pM) for 24 h. Data are shown as the mean ± SEM; *n* = 4–5. *P < 0.05, **P < 0.01, ***P < 0.001 vs. Scrambled siRNA.

Orai1 is an ion channel subunit that is a key mediator of SOCE in nonexcitable cells (11). To investigate the role of Orai1-mediated SOCE in the effect of PTH on HUVEC proliferation, we first treated HUVECs with 100 pM PTH for 24 h and determined SOCE activity. As shown in Figures 2A,B, SOCE was evoked by 2 μM thapsigargin (TG), an endoplasmic reticulum Ca^2+^-ATPase inhibitor, to deplete endoplasmic reticulum Ca^2+^ stores in a Ca^2+^-free solution, which was achieved by the addition of 2 mM Ca^2+^ to the extracellular bath solution. The Ca^2+^ influx was significantly increased in PTH-treated cells compared with control cells, and this effect was inhibited by both the Orai nonspecific inhibitor BTP2 and Orai1-specific small interfering RNA (siRNA) (Figures 2B–E). The ability of the siRNA to reduced Orai1 expression was confirmed by Western blotting (Figures 2F,G). In addition, PTH treatment for 24 h significantly enhanced basal [Ca^2+^]_i_ in HUVECs (Supplementary Figure 2). We also investigated whether the PTH-induced proliferation and viability increase of HUVECs was mediated by Orai1. Our data showed that compared with the transfection of scrambled siRNA, Orai1-specific siRNA Orai1 protein knockdown markedly reduced the proliferation and viability of HUVECs in both control and PTH-treated groups (Figure 2H). No significant difference was found between the control and PTH-treated groups in Orai1-specific siRNA transfected cells. These results suggested that PTH increased SOCE activity and proliferation of HUVECs via Orai1-mediated Ca^2+^ signaling.

### PTH-Induced Transcriptome Profile Change of HUVECs

**Figure 3:**
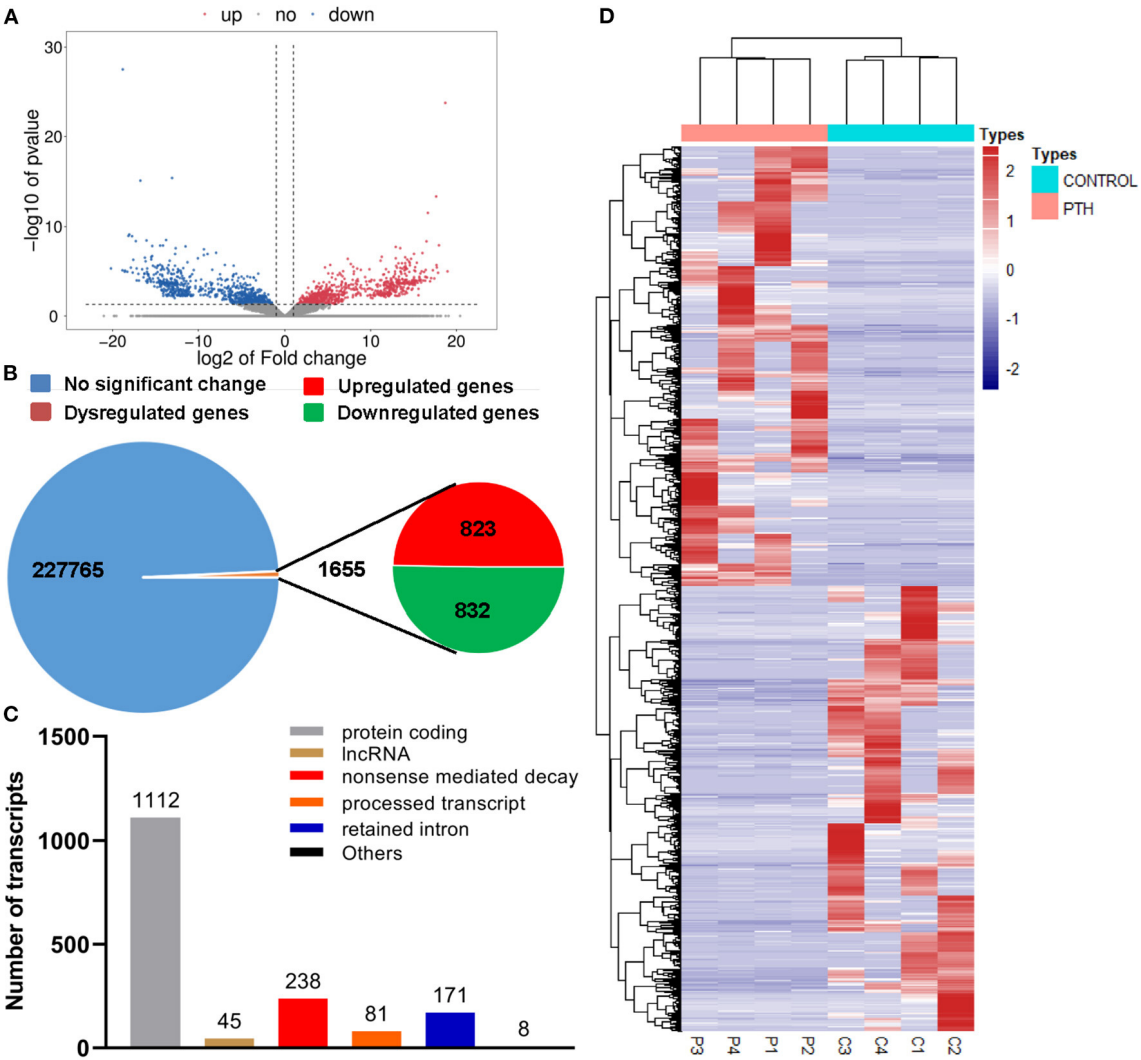
Effect of parathyroid hormone (PTH) on the transcriptome profile of human umbilical vein endothelial cells (HUVECs). **(A,B)** Volcano plot **(A)** and statistical graphs **(B)** of differentially expressed genes (DEGs). **(C)** Number of differentially expressed transcripts. **(D)** Heat map of the DEGs. Red represents highly expressed genes; blue, low expression. DEGs were identified with corrected *P* < 0.05 and absolute fold change ≥2. C1-4 represents the control group; P1-4, the PTH-treated (100 pM, 24 h) group.

To identify the underlying molecular mechanisms associated with PTH effects on HUVECs, we assessed gene expression changes using RNA sequencing (RNA-seq) techniques. As shown in the volcano plot and statistical graphs of Figures 3A,B, we detected 1,655 genes, including 823 upregulated genes and 832 downregulated genes, with fold changes ≥2 in the PTH-treated group compared with the control group. Among these differentially expressed genes (DEGs), 1,112 were protein-coding RNAs, 45 were long non-coding RNAs (lncRNA), and 498 were to be experimentally confirmed, nonsense-mediated decay, and processed transcripts (Figure 3C). The heat map in Figure 3D shows that the expression profiles of select genes were that significantly changed in HUVECs.

### Kyoto Encyclopedia of Genes and Genomes (KEGG) Pathway Analysis

**Figure 4:**
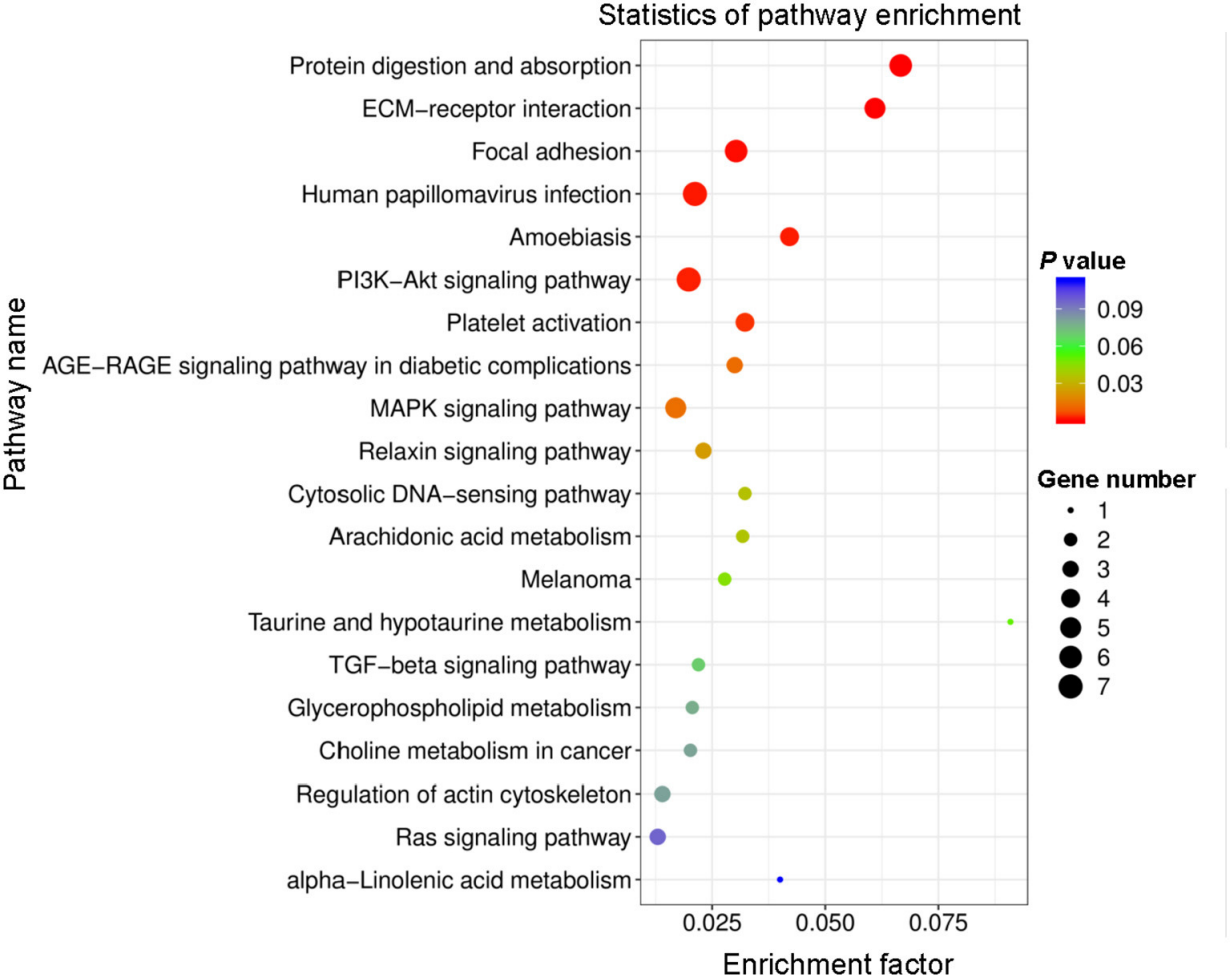
Top 20 enriched Kyoto Encyclopedia of Genes and Genomes (KEGG) pathway terms of differentially expressed genes (DEGs). Rich factor represents the percentage of DEGs in the KEGG relative to the identified genes in that classification. Circle size and color indicate the number of genes and *P*-value, respectively.

**Figure 5:**
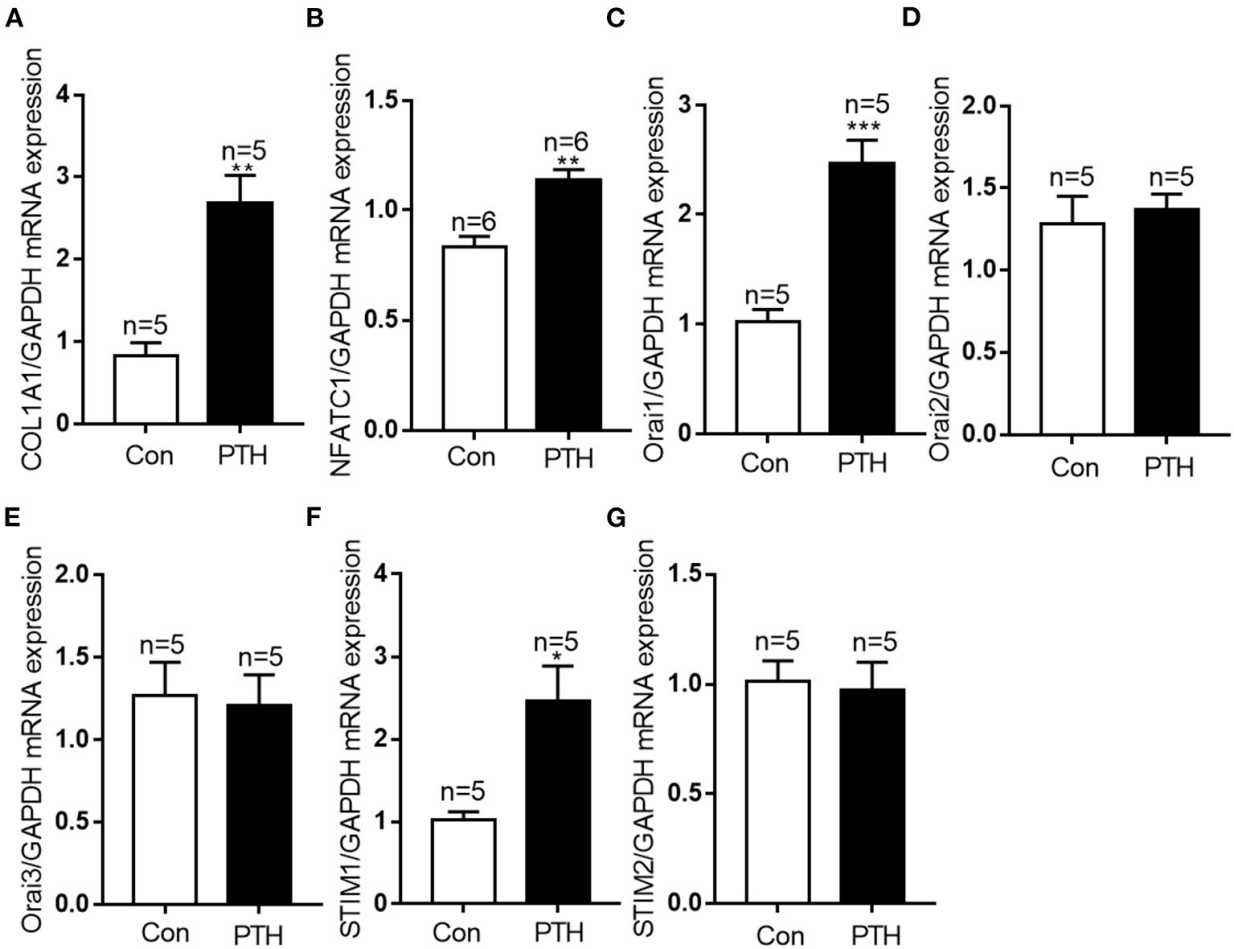
Effect of parathyroid hormone (PTH) on mRNA expressions of *COL1A1, NFATC1, Orai1, Orai2, Orai3, STIM1* and *STIM2* in human umbilical vein endothelial cells (HUVECs). **(A–G)** After the treatment with vehicle control or 100 pM PTH for 24 h, alterations in **(A)** *COL1A1*, **(B)** *NFATC1*, **(C)** *Orai1*, **(D)** *Orai2*, **(E)** *Orai3*, **(F)** *STIM1*, **(G)** *STIM2* mRNA levels were detected by qPCR assays in HUVECs. Messenger RNA expression was normalized to that of GAPDH. Data are shown as the mean ± SEM; *n* = 5 – 6. *P < 0.05, **P < 0.01, ***P < 0.001 vs. Con.

All DEGs were assessed using KEGG enrichment analysis. The top 20 signaling pathways ranked by *P* value are shown in Figure 4. Among them, the top three signaling pathways were protein digestion and absorption, ECM-receptor interaction, and focal adhesion. Many studies have reported that dysregulation of focal adhesion is closely associated with endothelial dysfunction (23, 24), such as migration and hyperproliferation. Therefore, further analyses were focused on the focal adhesion pathway. We found six DEGs, including *COL1A1, PDGFRA, COL6A2, COL6A3, COL1A2* and *THBS2*, that were involved in the focal adhesion pathway. Of these six DEGs, the expression level of *COL1A1* was highest. Previous studies have reported that the *COL1A1* gene plays important roles in cell migration and proliferation (25, 26). Therefore, we next assessed the mRNA expression level of *COL1A1* by using quantitative polymerase chain reaction (qPCR) assays. Our data showed that the level of *COL1A1* mRNA expression was significantly increased in the PTH-treated group compared with the control group (Figure 5A). We also investigated the involvement of *NFATC1* in cell migration and proliferation and found that *NFATC1* was significantly increased in the PTH-treated groups (Figure 5B). In addition, our data also showed that the mRNA expression levels of Orai1 (Figure 5C) and STIM1 (Figure 5F) were significantly increased, but Orai2 (Figure 5D), Orai3 (Figure 5E) and STIM2 (Figure 5G) were not changed in the PTH-treated HUVECs compared with the control group. These results suggested that *COL1A1* and *NFATC1* may be important downstream signaling molecules of PTH-induced migration and proliferation of HUVECs.

### Role of Orai1-Mediated Ca^2+^ Signaling in NFATC1 Translocation in PTH Effects on HUVECs

**Figure 6:**
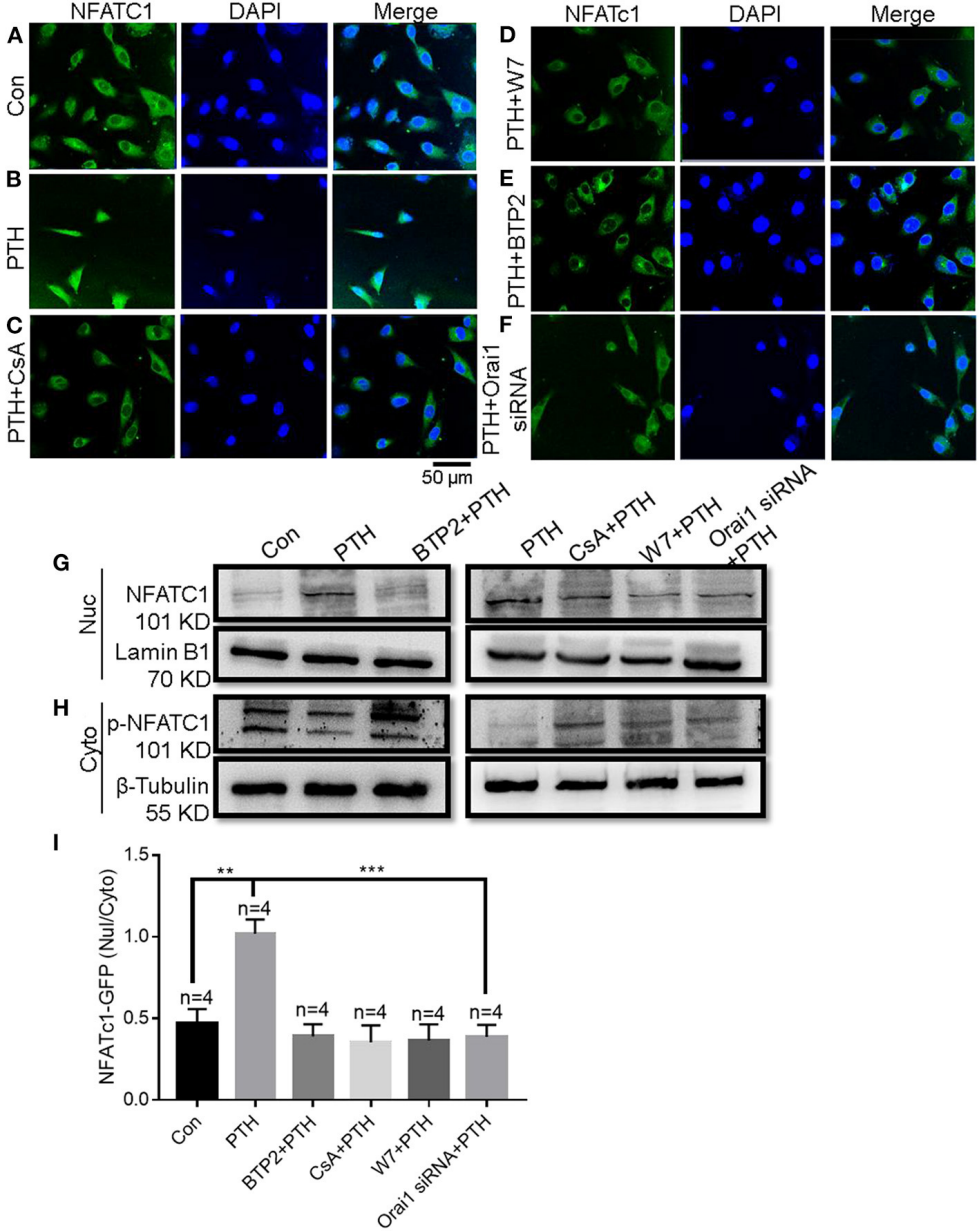
Role of Orai1-mediated store-operated Ca^2+^ entry (SOCE) in parathyroid hormone (PTH)-induced NFAT nuclear translocation in human umbilical vein endothelial cells (HUVECs). **(A–F)** Representative confocal microscopy images and summary data **(I)** showing NFATC1 distribution in HUVECs treated for 24 h with **(A)** vehicle control, **(B)** 100 pM PTH, **(C)** PTH + CsA (calcineurin inhibitor), **(D)** PTH + W7 (calmodulin antagonist), **(E)** PTH + BTP2 (an Orai nonspecific inhibitor) or **(F)** PTH + Orai1 siRNA transfection. Green fluorescence indicates NFATC1; Blue, 4′,6-diamidino-2-phenylindole (DAPI) indicates nuclei. Data are shown as the mean ± SEM; *n* = 4. **P < 0.01, ***P < 0.001 vs. Con or PTH analyzed by one-way analysis of variance followed by Dunnett's multiple comparisons test. **(G,H)** Representative Western blot images showing fractionation assay results indicating the presence of p-NFATC1 in the cytoplasmic [Cyto, **(H)**] and NFATC1 in the nuclear [Nuc, **(G)**] extracts under the same treatment conditions as for confocal microscopy analyses. Lamin B1 is a nuclear marker; β-Tubulin is a cytoplasmic marker. **(I)** Summary data showing the ratio of green fluorescence intensity of NFATc-GFP in the nuclear (Nuc)/cytoplasmic (Cyto).

NFAT acts as a Ca^2+^-sensitive transcription factor, integrating Ca^2+^ signaling with other pathways involved in diverse cellular functions, such as cell survival, proliferation, migration, invasion, and angiogenesis (27). After dephosphorylation by calcineurin, NFAT proteins translocate from the cytosol to the nucleus, where they bind to promoter regions to turn on the expression of numerous genes (14, 28, 29). To explore the effect of PTH on NFAT signaling in endothelial cells, we conducted immunofluorescence assays. As shown in Figures 6A–F,I, NFATC1 translocated to the cell nucleus in HUVECs treated with PTH, and this nuclear translocation was blocked by both the calcineurin inhibitor cyclosporin A (CsA; 1 μM) (Figure 6C) and by the selective calmodulin antagonist W7 (10 μM) (Figure 6D). These results suggested a pivotal role of PTH in activating calcineurin for NFATC1 dephosphorylation subsequently leading to nuclear translocation. We then either treated HUVECs with the SOCE inhibitor BTP2 (10 μM) (Figure 6E) or transfected HUVECs with Orai1-specific siRNA (Figure 6F) and found that NFAT nuclear translocation was inhibited in both treated groups. To confirm NFAT nuclear translocation, we also conducted fractionation experiments. Phosphorylated NFATC1 (p-NFATC1) remains in the cytosol, but dephosphorylated NFATC1 can translocate to the nucleus (14). The cytoplasmic and nuclear components were extracted from total HUVECs lysates. As shown in Figure 6G, in agreement with our immunofluorescence data, PTH elicited nuclear translocation of NFATC1 in HUVECs. The increased NFATC1 level in the nuclear fraction was inhibited by pretreating the cells with CsA, W7, BTP2 or Orai1-specific siRNA (Figure 6H). The level of p-NFATC1 protein expression was significantly decreased in the cytosol of HUVECs treated with PTH compared with controls, but this decrease was blocked by pretreating the cells with CsA, W7, BTP2 or Orai1-specific siRNA. These results indicated that Orai1-mediated Ca^2+^ influx activates the calmodulin/calcineurin/NFATC1 signaling pathway to induce NFATC1 nuclear translocation.

### PTH Regulates COL1A1 Expression by Enhancing NFATC1 Nuclear Translocation

**Figure 7:**
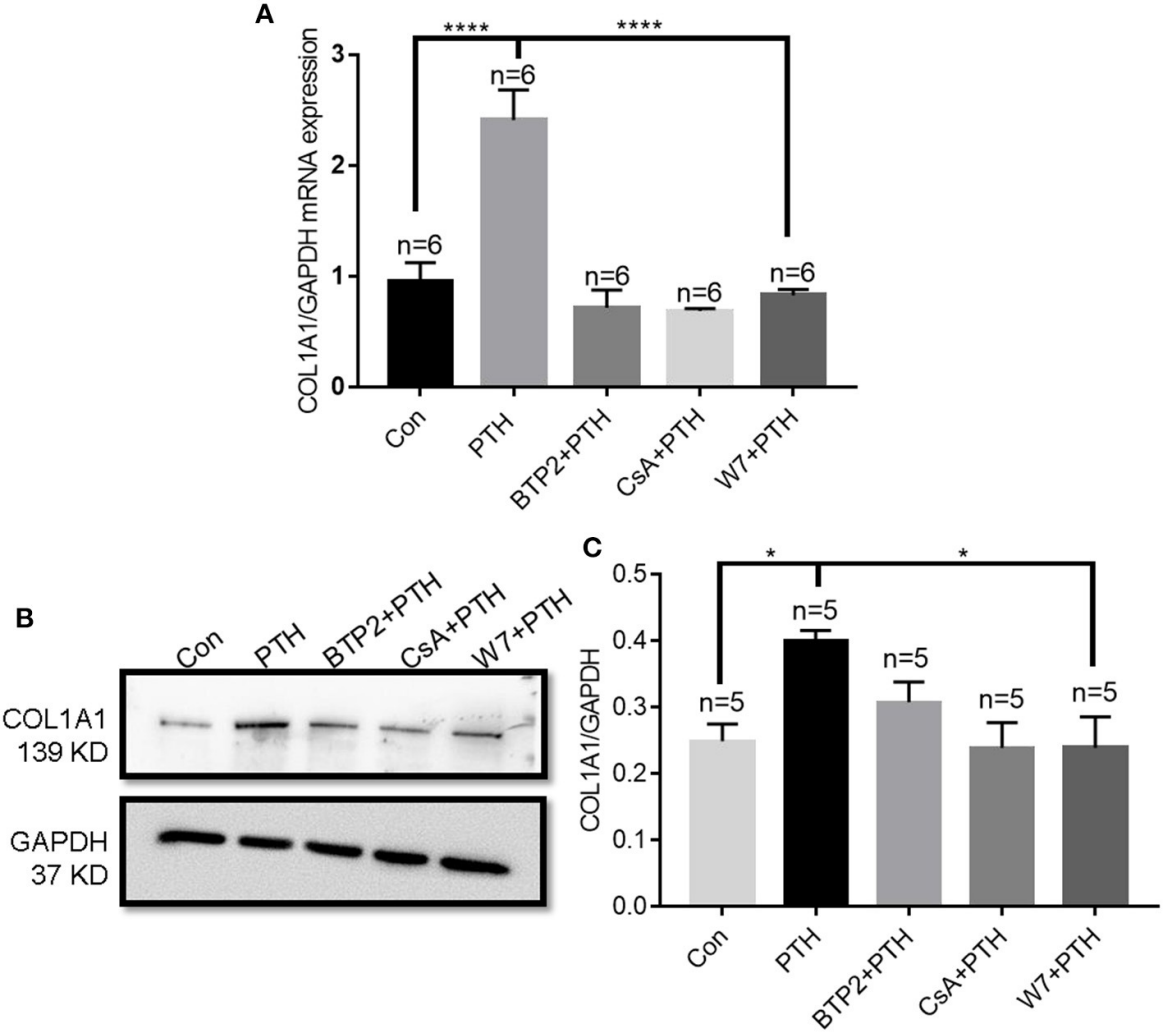
Effect of parathyroid hormone (PTH) on COL1A1 expression in human umbilical vein endothelial cells (HUVECs). **(A)** After HUVECs were treated with vehicle control, 100 pM PTH, PTH + BTP2 (an Orai nonspecific inhibitor), PTH + CsA (calcineurin inhibitor), or PTH + W7 (calmodulin antagonist) for 24 h, *COL1A1* mRNA levels were analyzed by qPCR assays. Messenger RNA expression was normalized to that of GAPDH. **(B,C)** Representative images **(B)** and summary data **(C)** showing COL1A1 protein expression in HUVECs after treatment with vehicle control, 100 pM PTH, PTH + BTP2, PTH + CsA or PTH + W7 for 24 h. GAPDH expression was referenced as the loading control. Data are shown as the mean ± SEM; *n* = 5–6. *P < 0.05, ****P < 0.0001 vs. Con or PTH in **(A)** and **(C)** analyzed by one-way analysis of variance followed by Dunnett's multiple comparisons test.

When NFATC1 translocates from the cytosol to the nucleus, it can bind to gene promoter regions and induce the expression of numerous genes (29). Therefore, we next investigated the role of the calmodulin/calcineurin/NFATC1 signaling pathway on the regulation of COL1A1 expression. We found that PTH treatment significantly increased both the mRNA and protein expression levels of COL1A1, and these increases were significantly blocked by treatment with CsA, W7, and BTP2 (Figure 7).

### Roles of COL1A1 in PTH-Increased HUVEC Migration and Proliferation

**Figure 8:**
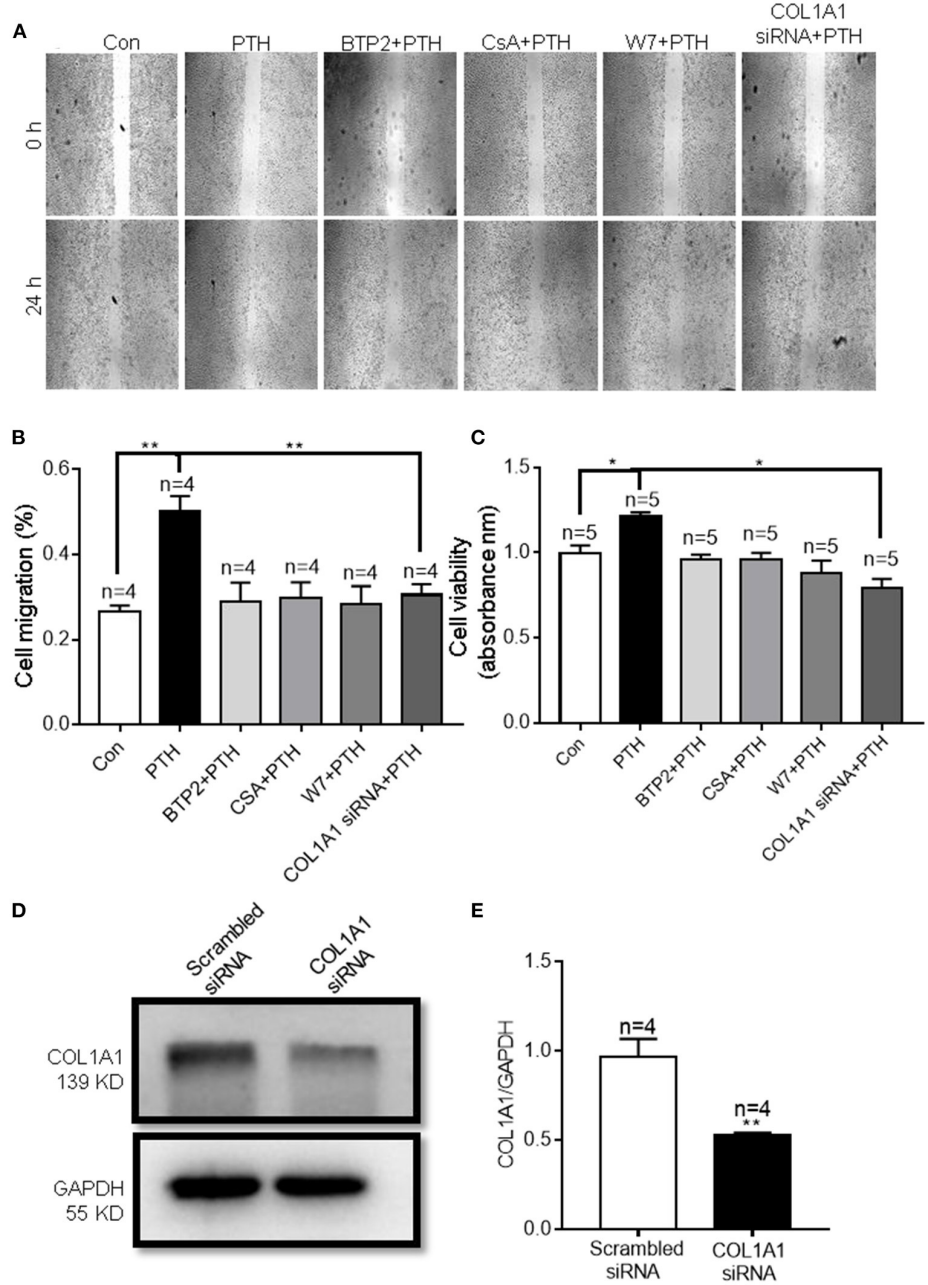
Effect of parathyroid hormone (PTH) on migration of human umbilical vein endothelial cells (HUVECs) **(A,B)**. **(A)** Representative images showing the migration of HUVECs treated with vehicle control (Con), 100 pM PTH, PTH + BTP2 (an Orai nonspecific inhibitor), PTH + CsA (calcineurin inhibitor), PTH + W7 (calmodulin antagonist), or PTH + *COL1A1* siRNA transfection. **(B)** Summary data showing the percentage of cells that migrated during 24 h. **(C)** Summary data showing the viability of HUVECs treated with vehicle control, 100 pM PTH, PTH + BTP2, PTH + CsA, PTH + W7, or PTH + *COL1A1* siRNA transfection for 24 h. Data are shown as the mean ± SEM; *n* = 4–5. *P < 0.05, **P < 0.01 vs. Con or PTH analyzed by one-way analysis of variance followed by Dunnett's multiple comparisons test. **(D,E)** Representative images and summary data showing the effect of scrambled siRNA or *COL1A1* siRNA transfection on COL1A1 expression in HUVECs. GAPDH was used as a loading control. Data are shown as the mean ± SEM; *n* = 4–5. **P < 0.01 vs. Scrambled siRNA.

**Figure 9:**
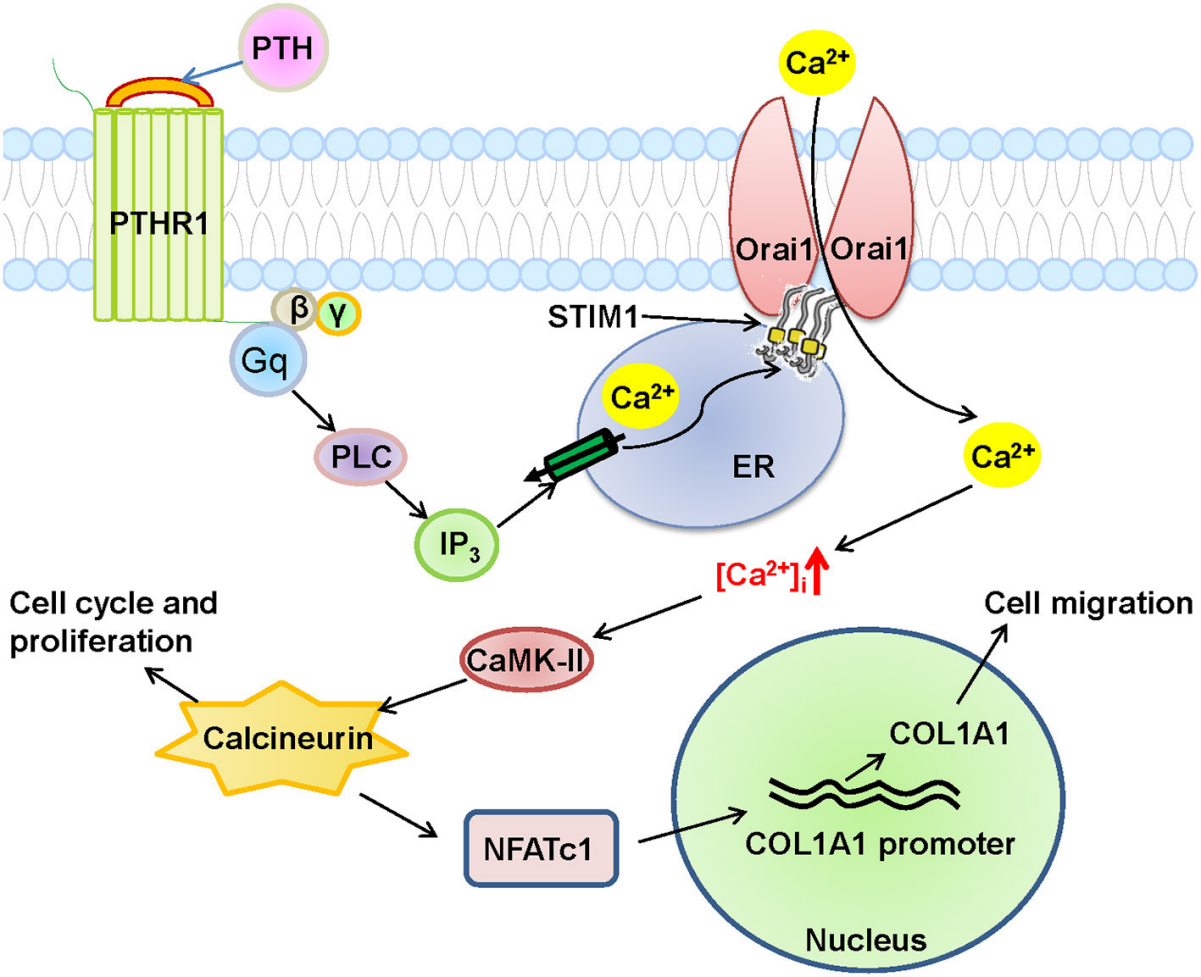
Schematic diagram of Orai1-mediated store-operated Ca^2+^ entry (SOCE) signal transduction pathway in human umbilical vein endothelial cells (HUVECs) after parathyroid hormone (PTH) stimulation. In this proposed mechanism, PTH acts on its receptor PTHR1, a G-protein coupled receptor, to produce phospholipase C (PLC) and then inositol triphosphate (IP_3_), which stimulates its receptor to deplete Ca^2+^ stores in the endoplasmic reticulum (ER), leading to the opening of the Orai1 channel in HUVECs to induce extracellular Ca^2+^ influx via SOCE. The subsequent increase in intracellular Ca^2+^ ([Ca^2+^]_i_) causes the nuclear translocation of NFATC1 through the Ca^2+^/calmodulin-dependent kinase 2 (CaMK-II)/calcineurin signaling pathway, and eventually induces COL1A1 overexpression and HUVEC migration and proliferation.

COL1A1, an α1 chain of type I collagen and the main constituent of the extracellular matrix component in tumors, is essential for specific interactions between cell surface and transmembrane molecules and is involved in the control of cellular biological activities, such as cell differentiation, proliferation, adhesion, migration, invasion, and apoptosis (30, 31). To explore the effect of COL1A1 on cell migration, we conducted wound healing assays. As shown in Figures 8A,B, PTH significantly increased the percentage of migrating HUVECs compared with that in the control group. However, knockdown of COL1A1 expression by specific siRNA transfection or pretreatment with CsA, W7, or BTP2 markedly suppressed the PTH-induced increase in HUVEC migration (Figures 8A,B). To determine whether PTH promoted the proliferation of HUVECs, we used the CCK-8 assay to assess HUVEC viability. As shown in Figure 8C, PTH significantly increased the proliferation of HUVECs compared with that in the control group. Similar to the cell migration assay results, the PTH-induced increase in HUVEC proliferation was significantly inhibited by COL1A1-specific siRNA transfection or by pretreatment with CsA, W7, or BTP2 (Figure 8C). The effect of COL1A1-specific siRNA on COL1A1 protein expression was also detected by Western blotting (Figures 8D,E). Together, these results indicated that PTH treatment enhanced HUVEC migration and proliferation through the Orai1/calmodulin/calcineurin/NFATC1/COL1A1 signaling pathway (Figure 9).

## Discussion

Endothelial dysfunction is a critical risk factor for vascular diseases and plays a key role in atherosclerosis, hypertension, diabetes and CVD (29, 32). PTH is associated with several CVDs (5, 9, 33). However, the molecular mechanism by which PTH regulates endothelial cell proliferation and migration and endothelial dysfunction, leading to vascular disease, remains unclear. In this study, we showed that PTH regulates SOCE activity, and we investigated the functional role of this regulation in endothelial cells. Our main findings are as follows: (1) The expression levels of Orai1 channel protein and HUVEC cell proliferation were significantly increased in HUVECs treated with PTH, whereas the expression levels of the Orai2 and Orai3 subunits remained unchanged. (2) The activity of SOCE was significantly increased in HUVECs treated with PTH, and knockdown of Orai1 protein in HUVECs suppressed SOCE activity and cell viability. (3) RNA-seq analysis revealed 1655 DEGs in PTH-treated HUVECs compared with control cells, including 823 upregulated genes and 832 downregulated genes. KEGG pathway enrichment analysis showed that the focal adhesion pathway was upregulated in the PTH-treated group, and the expression levels of two key genes, *COL1A1* and *NFATC1*, whose translated proteins are involved in cell migration and proliferation, were also upregulated. (4) Nuclear translocation of NFATC1 was evoked by PTH, and this translocation was blocked by CsA, W7, BTP2, and Orai1 knockdown in HUVECs. (5) The expression levels of COL1A1 mRNA and protein in HUVECs were enhanced by PTH administration, and this enhancement was blocked by CsA, W7 and BTP2. (6) HUVEC migration and proliferation were increased by PTH, and the increases in these processes were blocked by CsA, W7, BTP2 and *COL1A1* siRNA transfection. Together, these data suggest that PTH increases SOCE activity and downstream NFATC1 nuclear translocation in HUVECs, leading to increased COL1A1 expression, which regulates HUVEC migration and proliferation (Figure 9). To our knowledge, this is the first study to report these important findings.

SOCE participates not only in relevant signaling pathways of physiological processes but also in various pathological processes. In fact, many studies have indicated that SOCE-related proteins, including Orais and STIM1, affect the vascular system in a variety of pathological conditions. The Orai1 protein, which is located in the endothelial cell plasma membrane, is involved in various dysfunctions of the endothelium (12, 29–31). For example, Orai1 and STIM1 mediate histamine-induced endothelial inflammation, and Orai1 is involved in endoplasmic reticulum stress–induced endothelial dysfunction (33, 34). Our study showed that Orai1 was involved in the PTH-induced increase in SOCE activity in HUVECs, whereas Orai2 and Orai3 did not participate. These findings indicate that different components of SOCE may contribute to different responses. Orai1 is likely a key mediator in PTH-induced endothelial dysfunction in CVD.

The calcineurin/NFAT signaling pathway is involved in the genomic regulation of many cellular processes, including the cell cycle, development, differentiation, and angiogenesis (35–37), and SOCE-mediated Ca^2+^ regulatory signaling is critical for NFAT activation and downstream signal transduction in a variety of autoimmune diseases (15, 38). But we still do not know the role of this pathway in CVD. In response to histamine stimulation, endothelial cells upregulate interleukin 8 production through the calcineurin/NFAT pathway, leading to endothelial inflammation. In the present study, we found that PTH also activated the NFATC1 pathway and increased COL1A1 expression through Orai1-mediated Ca^2+^ entry. Orai1 knockdown or calcineurin/NFAT pathway inhibitors reduced the PTH-induced NFATC1 nuclear translocation and the PTH-induced increase in COL1A1 expression. Studies from other groups have shown that COL1A1 participates in cell adhesion and motility in various diseases (39–41). Therefore, we used RNA-seq and bioinformatics analyses to find that COL1A1 transcriptome expression was increased in PTH-treated HUVECs (42, 43). COL1A1 has also been reported to be upregulated in osteoblasts via the calcineurin/NFAT signaling pathway (44). We found that the PTH-increased COL1A1 expression was also mediated via activation of the calcineurin/NFATC1 signaling pathway, thereby enhancing the migration and proliferation of HUVECs; COL1A1 knockdown or calcineurin/NFAT pathway inhibitors significantly reduced the migration and proliferation of HUVECs. Thus, our study suggests an Orai1/calmodulin/calcineurin/NFATC1/COL1A1 signaling pathway in PTH-increased endothelial cell migration and proliferation (Figure 9).

There are some limitations to the present study because SHPT-associated vascular disease is a complex process involving interactions of primary disease, hypercalcemia, hyperphosphatemia, and parathyroid hormone abnormalities (21, 45). Therefore, further analyses using animal models is suggested, and potential therapeutic applications should be evaluated.

## Conclusion

In conclusion, this study is the first, to our knowledge, to demonstrate that PTH increased Orai1 protein expression and Orai1-mediated SOCE activity in HUVECs, leading to the nuclear translocation of NFATC1 to subsequently enhance COL1A1 expression and COL1A1-mediated migration and proliferation of HUVECs. Our results suggest that Orai1 and the downstream calmodulin/calcineurin/NFATC1/COL1A1 signaling pathway may play an important role in PTH-induced endothelial dysfunction and may be key potential therapeutic targets for preventing or treating SHPT-induced CVD.

## Data Availability Statement

The sequenced data presented in the study are deposited in the NCBI repository, accession number PRJNA807401.

## Author Contributions

SW, LX and BS contributed to the design of the study. SW, LX, HS, YW, and ZL performed the experiments. LZ, BS, YL, and KW supervised the project. SW, BS and YF wrote the manuscript. All authors participated in revising the article and approved the final manuscript.

## Funding

The current work was supported by the Natural Science Foundation of China (Grant No. 82171127), the Natural Science Foundation of Anhui Province (Grant No. 1808085MH252), the Discipline Construction Project of Anhui Medical University (Grant No. 2021lcxk007), the Anhui Provincial Institute of Translational Medicine (Grant No. 2021zhyx-C40), and the Project Funded by Scientific Research Platform and Base Upgrading Plan of Anhui Medical University (2021xkjT048).

## Conflict of Interest

The authors declare that the research was conducted in the absence of any commercial or financial relationships that could be construed as a potential conflict of interest.

## Publisher's Note

All claims expressed in this article are solely those of the authors and do not necessarily represent those of their affiliated organizations, or those of the publisher, the editors and the reviewers. Any product that may be evaluated in this article, or claim that may be made by its manufacturer, is not guaranteed or endorsed by the publisher.
