# Supplementary material for: Parathyroid Hormone Promotes Human Umbilical Vein Endothelial Cell Migration and Proliferation Through Orai1-Mediated Calcium Signaling
Source: Front Cardiovasc Med. 2022 Mar 16;9:844671. doi: 10.3389/fcvm.2022.844671 (PMC8965836; doi:10.3389/fcvm.2022.844671)
Supplement: Supplementary file 1 [file Data_Sheet_1.PDF]

# Supplementary Figures

Supplemental Methods

$[Ca^{2+}]_i$ measurement:

$[Ca^{2+}]_i$ measurements were performed as previously described [1]. Briefly, HUVECs were seeded on round glass cover slips placed in 12-well plates and treated with PTH (100 pM) for 24 h before measurement. The cells were incubated for 30 min at 37 °C with 2 μM Fura-2/AM and 0.02 % pluronic F-127 (Invitrogen) in the culture media. Intracellular Ca^{2+} stores were depleted using 2 μM TG in a Ca^{2+}-free saline solution (OPSS, 140 mM NaCl, 5 mM KCl, 2 mM $CaCl_2$, 1 mM $MgCl_2$, 10 mM glucose, and 5 mM HEPES, pH 7.3 to 7.4 adjusted with NaOH). Application of 2 mM Ca^{2+} to the medium evoked Ca^{2+} influx. Imaging data acquisition and analyses on both microscope systems were accomplished using MetaFluor software. Fura-2 data were expressed as the ratio of 340/380.

Western blotting:

Total protein was extracted from HUVECs after PTH treatment (1, 10, 100 pM) for 24 h. Protein-transferred membranes were incubated with the primary antibodies rabbit anti-PTHR1 antibody (1:1000 dilution; Sangon Biotech, China) at 4 °C for 24 h. The proteins were then treated with goat anti-rabbit IgG horseradish peroxidase–conjugated secondary antibody (1:5000 dilution, Elabscience Biotechnology, China) at room temperature for 1 h. The protein signal was detected using an ECL detection system (Peiqing Technology, China). The optical density of each blot was normalized to that of GAPDH and expressed as relative optical density. The blot images were analyzed using ImageJ (National Institutes of Health, Bethesda, Maryland).

Supplementary Figures

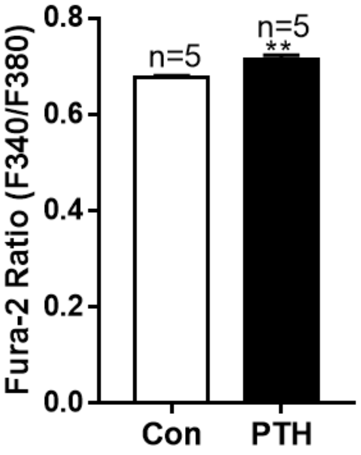

Supplementary Figure 1. Effect of parathyroid hormone (PTH) on intracellular Ca^{2+} concentration in human umbilical vein endothelial cells (HUVECs). Summary data showing the Fura-2 fluorescence ratio (F340/F380) in HUVECs after PTH treatment for 24 h. Data are shown as the mean ± SEM; n = 5. ***P* < 0.01, PTH vs. Control (Con).

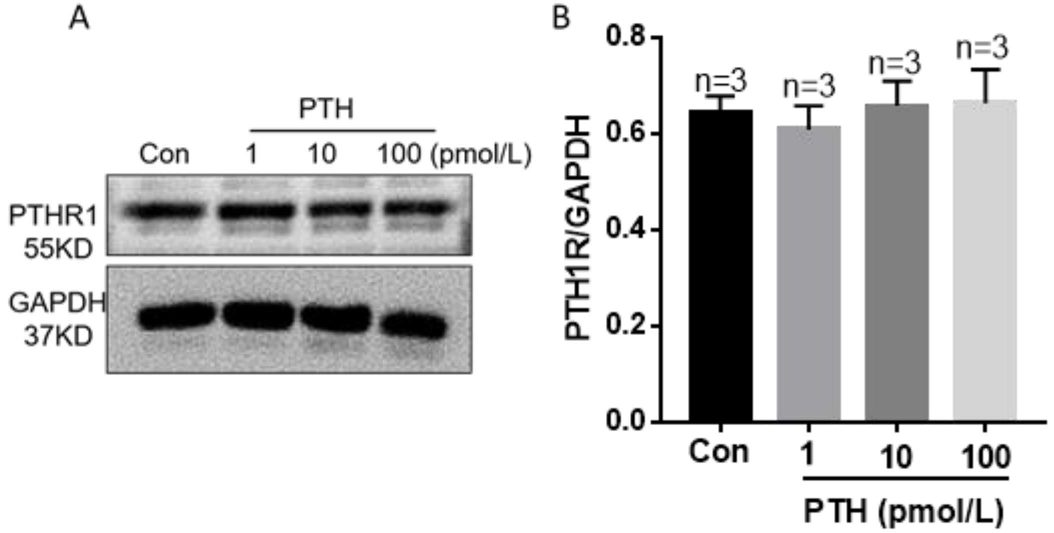

Supplementary Figure 2. Effects of parathyroid hormone (PTH) on the expression level of PTHR1 in human umbilical vein endothelial cells (HUVECs). (A-B) Representative Western blot images (A) and summary data (B) showing PTHR1 expression level change in HUVECs after PTH treatment (1, 10, 100 pM) for 24 h compared with the vehicle control (Con). Data are shown as the mean ± SEM; n = 3. ***P* < 0.01 vs. Con analyzed by one-way analysis of variance followed by Dunnett's multiple comparisons test.

References for Supplemental Data

1. J. Guo, R. Zhao, M. Zhou, J. Li, X. Yao, J. Du, J. Chen and B. Shen, Cell Commun Signal **18** (1), 138 (2020). DOI: 10.1186/s12964-020-00560-7
