# Supplementary material for: Parathyroid Hormone Promotes Human Umbilical Vein Endothelial Cell Migration and Proliferation Through Orai1-Mediated Calcium Signaling
Source: Front Cardiovasc Med. 2022 Mar 16;9:844671. doi: 10.3389/fcvm.2022.844671 (PMC8965836; doi:10.3389/fcvm.2022.844671)

Original Western blot 1

S1

Orai1
50 KD

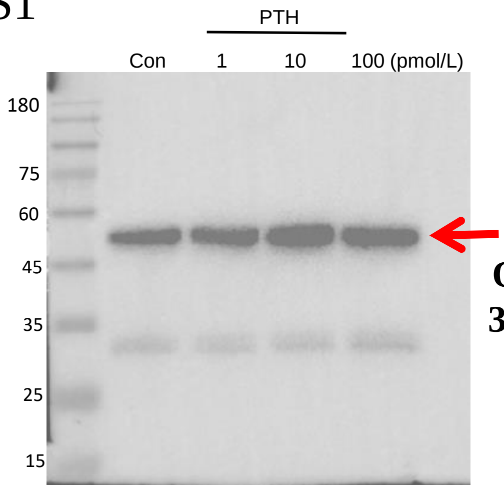

S2

Orai2
36 KD

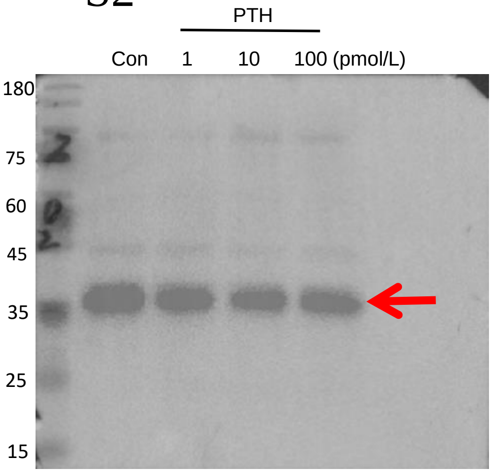

S3

Orai3
34 KD

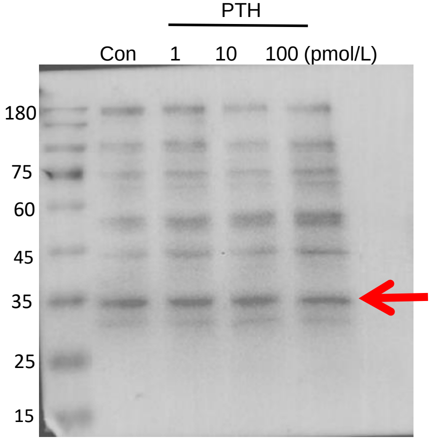

S4

STIM1
77 KD

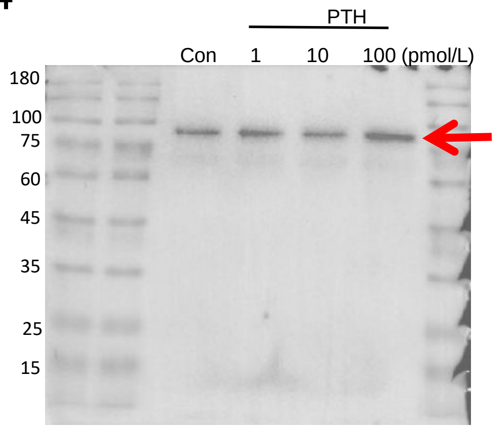

S5

β-Tubulin
55 KD

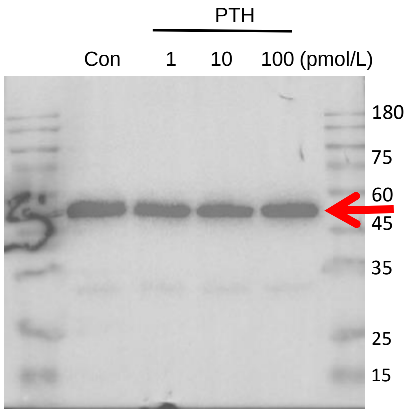

Original Western blot 2

S6

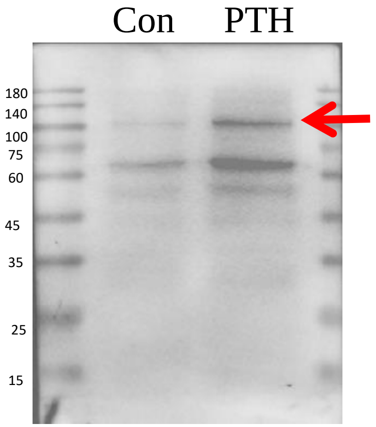

NFATC1
101 KD

S7

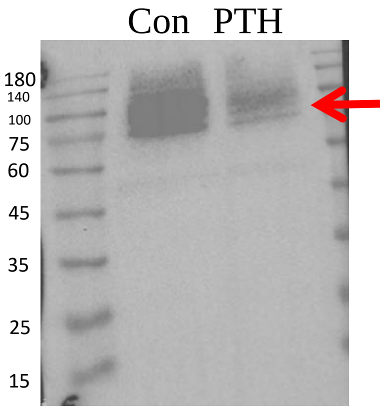

p-NFATC1
101 KD

S8

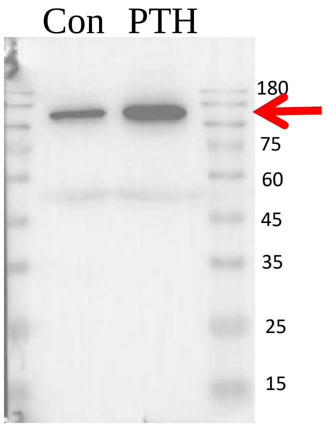

COL1A1
139 KD

S9

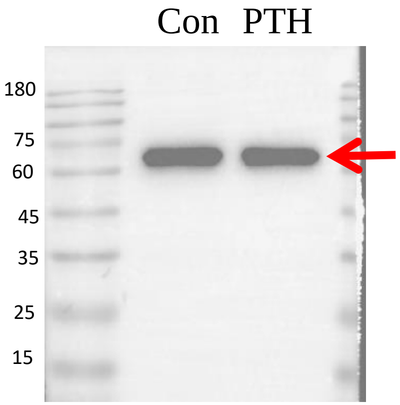

Lamin B1
70 KD

S10

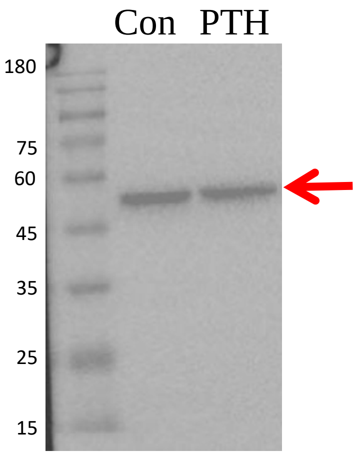

β-Tubulin
55 KD

S11

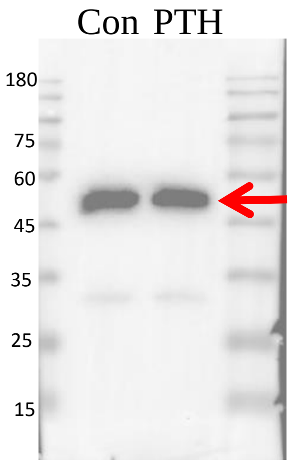

β-Tubulin
55 KD

Original western blot 3

S12

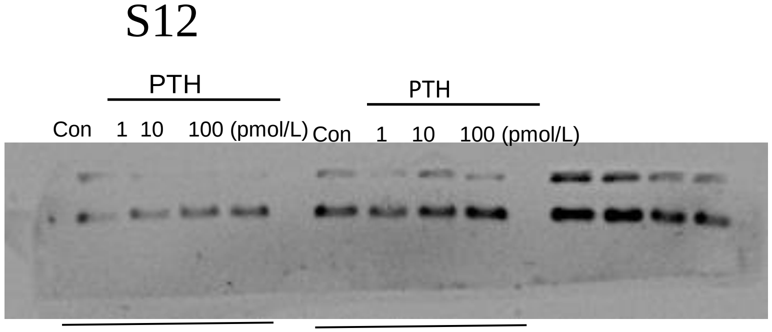

S13

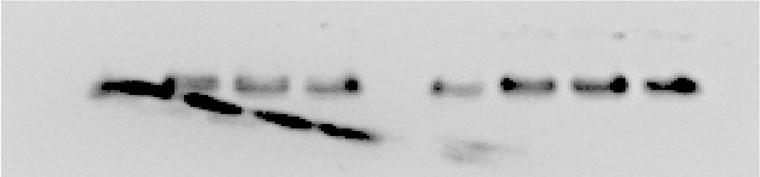

S14

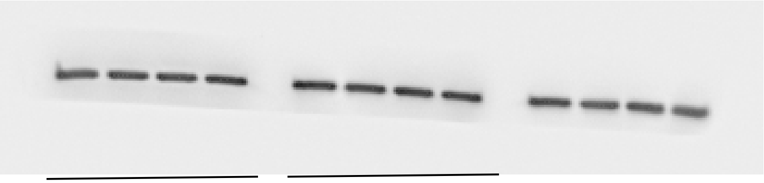

S15

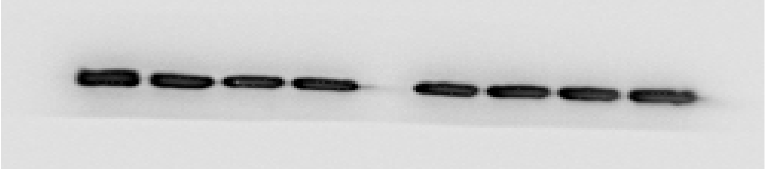

Original Western blot 4

S16

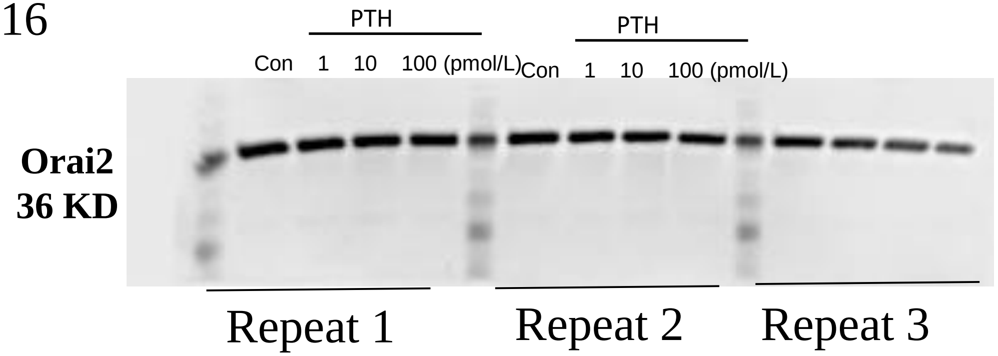

S17

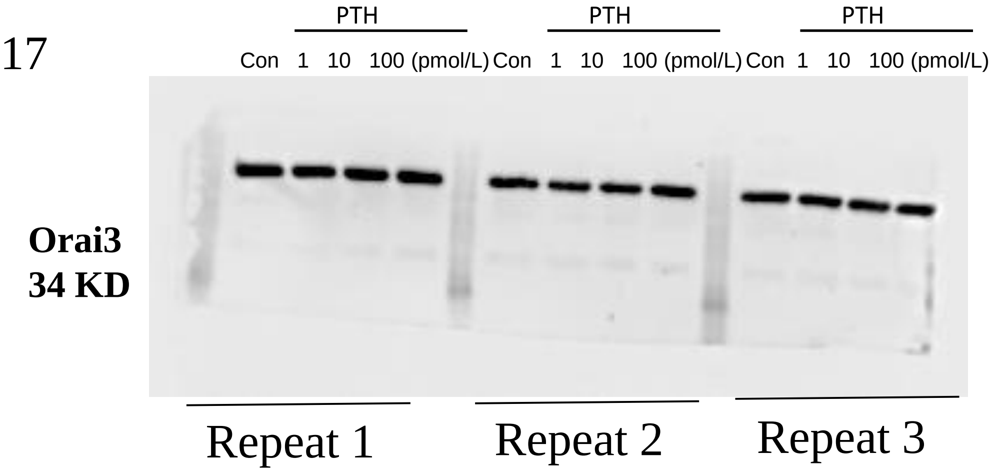

S18

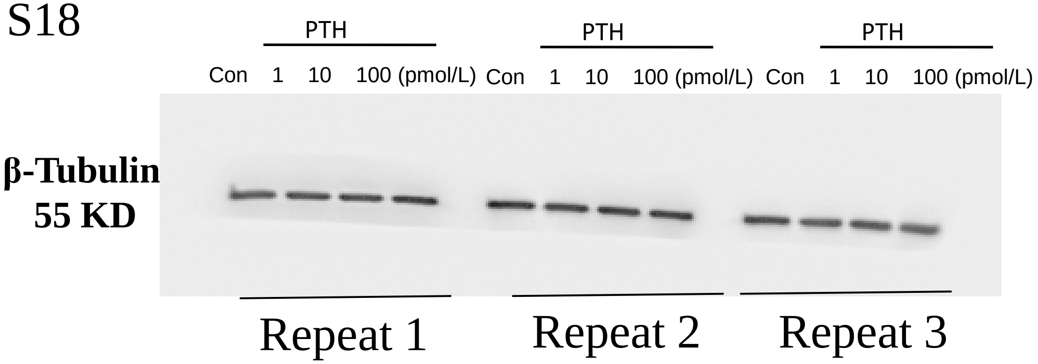

Original Western blot 5

S19

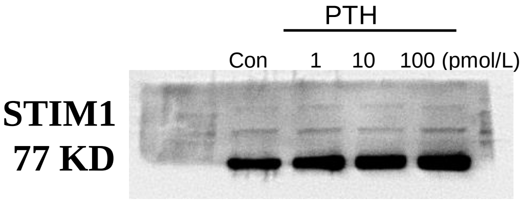

Repeat1

S20

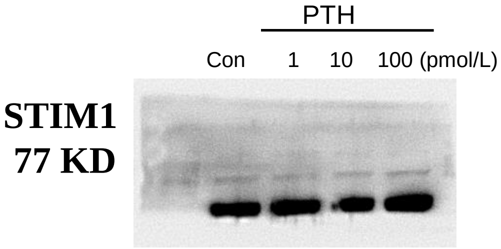

Repeat 2

S21

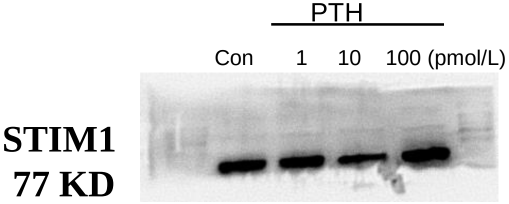

Repeat 3

S22

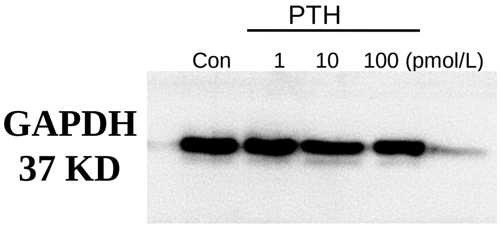

Repeat1

S23

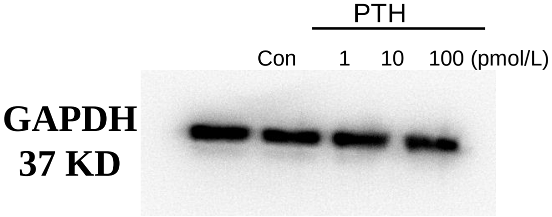

Repeat 2

S24

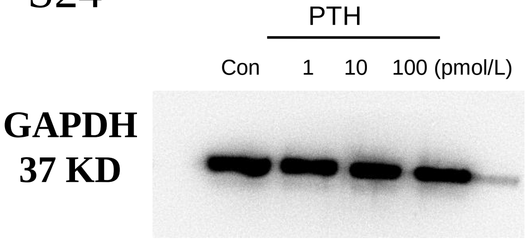

Repeat 3

Original Western blot 6

S25

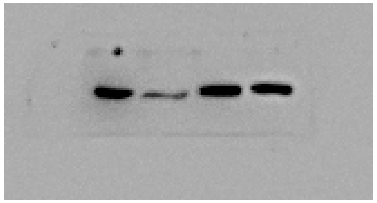

S26

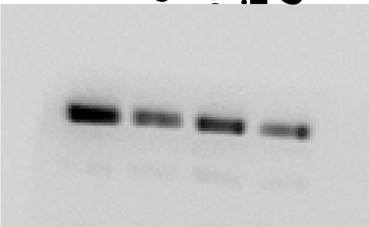

S27

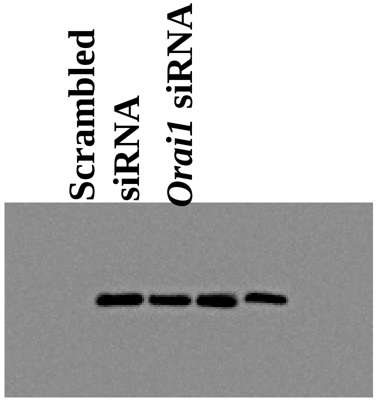

S28

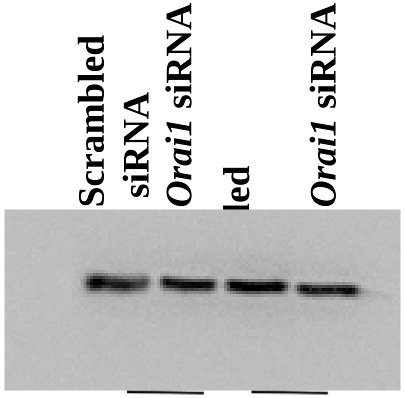

Original Western blot 7

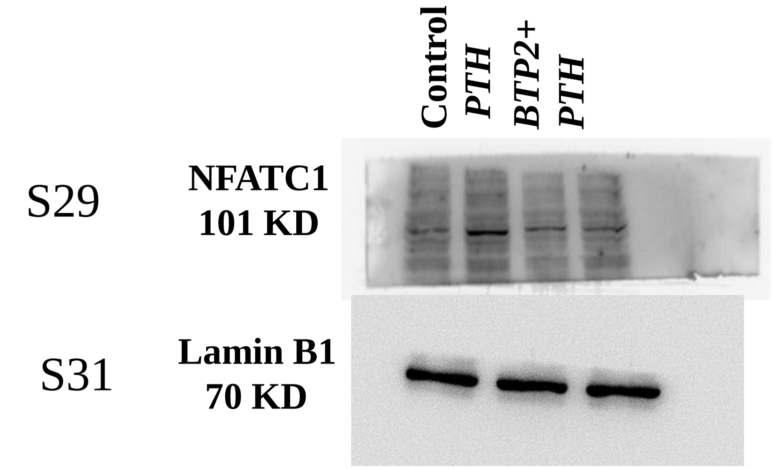

Repeat1

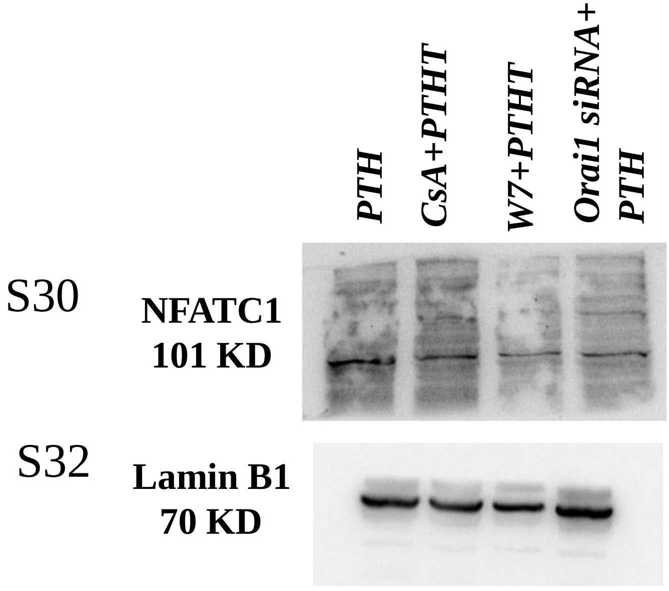

Repeat1

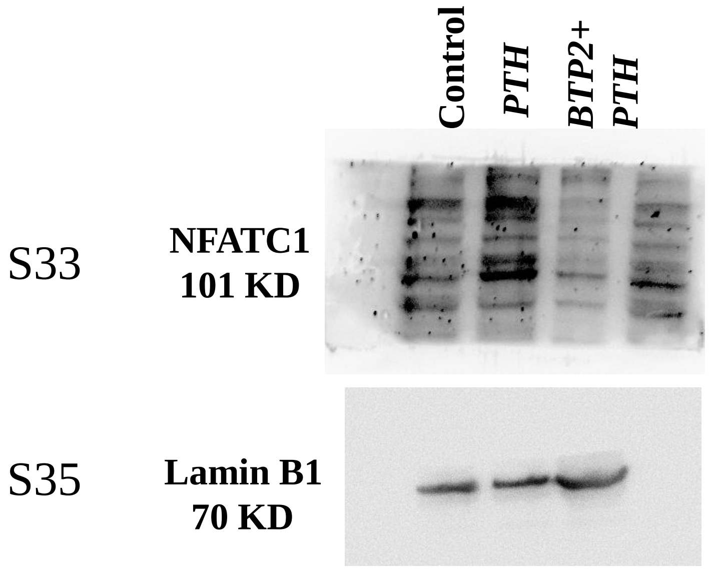

Repeat2

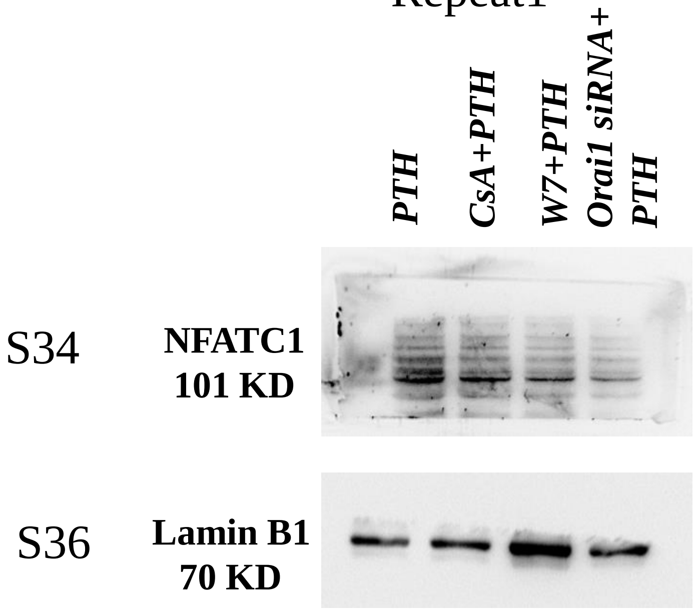

Repeat2

Original Western blot 8

S37 p-NFATC1 101 KD

S39 β-Tublin 55 KD

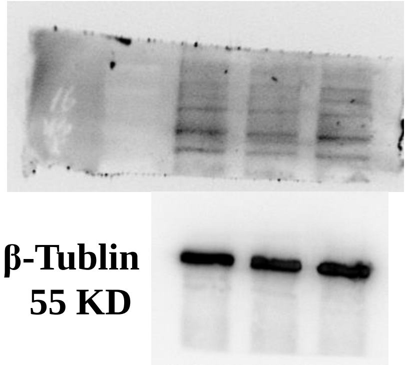

Repeat 1

S38 p-NFATC1 101 KD

S40 β-Tublin 55 KD

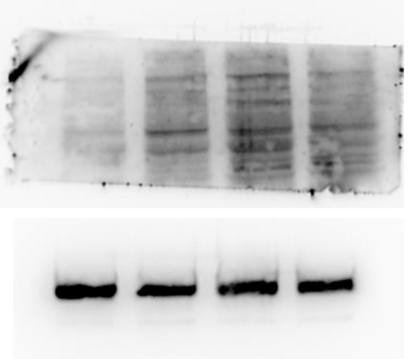

Repeat 1

S41 p-NFATC1 101 KD

S43 β-Tublin 55 KD

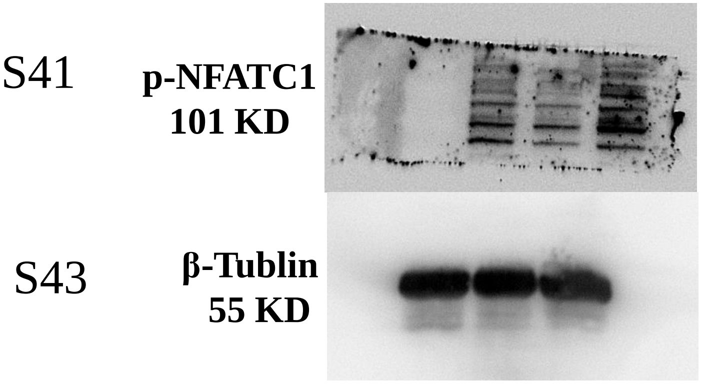

Repeat 2

S42 p-NFATC1 101 KD

S44 β-Tublin 55 KD

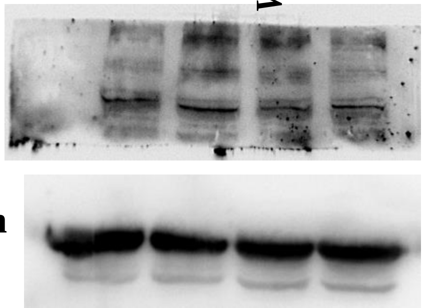

Repeat 2

Original Western blot 9

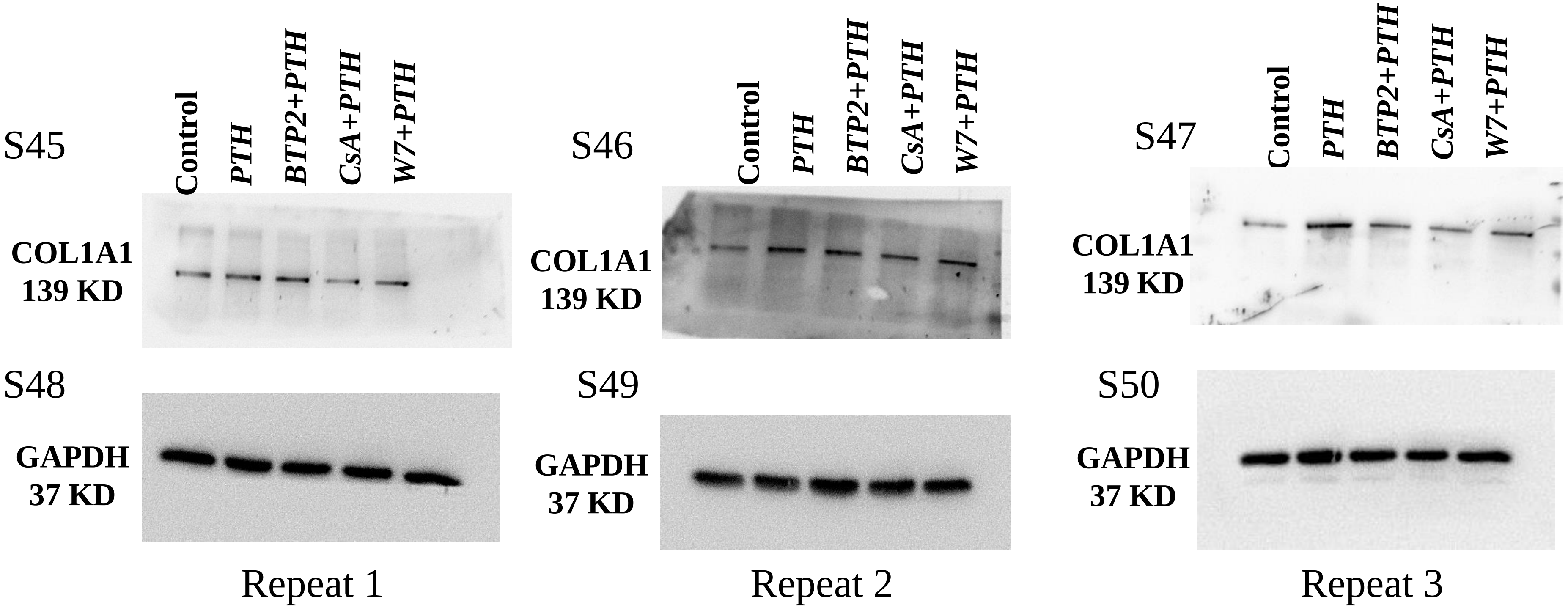

Original Western blot 9

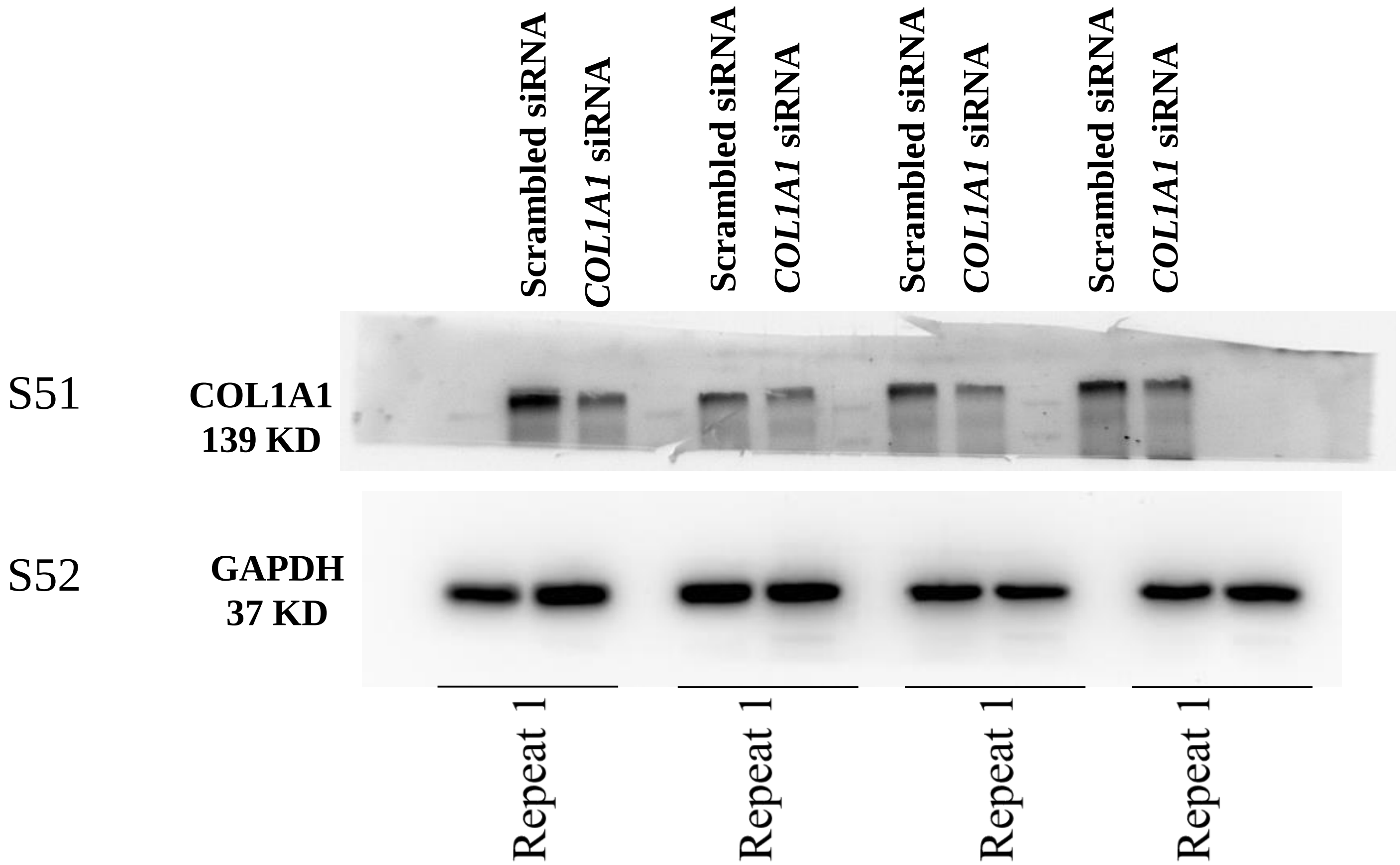

Supplement: Supplementary file 2 [file Data_Sheet_2.PDF]
